# LIVECell—A large-scale dataset for label-free live cell segmentation

**DOI:** 10.1038/s41592-021-01249-6

**Published:** 2021-08-30

**Authors:** Christoffer Edlund, Timothy R. Jackson, Nabeel Khalid, Nicola Bevan, Timothy Dale, Andreas Dengel, Sheraz Ahmed, Johan Trygg, Rickard Sjögren

**Affiliations:** 1 Sartorius Corporate Research, Umeå, Sweden; 2 Sartorius, BioAnalytics, Royston, UK; 3 Deutsches Forschungszentrum für Künstliche Intelligenz, GmbH (DFKI), Saarbrücken, Germany; 4 Computational Life Science Cluster (CLiC), Umeå University, Umeå, Sweden

**Keywords:** Research data, Cell biology, Technology

## Abstract

Light microscopy combined with well-established protocols of two-dimensional cell culture facilitates high-throughput quantitative imaging to study biological phenomena. Accurate segmentation of individual cells in images enables exploration of complex biological questions, but can require sophisticated imaging processing pipelines in cases of low contrast and high object density. Deep learning-based methods are considered state-of-the-art for image segmentation but typically require vast amounts of annotated data, for which there is no suitable resource available in the field of label-free cellular imaging. Here, we present LIVECell, a large, high-quality, manually annotated and expert-validated dataset of phase-contrast images, consisting of over 1.6 million cells from a diverse set of cell morphologies and culture densities. To further demonstrate its use, we train convolutional neural network-based models using LIVECell and evaluate model segmentation accuracy with a proposed a suite of benchmarks.

## Abstract (web summary)

The LIVECell dataset comprises annotated phase-contrast images of over 1.6 million cells from different cell lines during growth from sparse seeding to confluence for improved training of deep learning-based models of image segmentation.

## Main

Quantitative imaging offers unequaled spatial and temporal resolution when measuring biological phenomena, which has led to its wide use in cell biology and biomedical research. Two-dimensional (2D) cell monolayer models of mammalian cells are a cornerstone of cellular based research due to well-established and reproducible protocols. The low dimensional complexity of 2D cultures readily facilitates experimental methods, including imaging. In particular, cellular assays are an accessible medium to obtain physiologically relevant data from images, allowing quantification of the effects of interventions on cell count, proliferation, morphology, migration, cellular interactions and when coupled with fluorescence imaging, protein expression dynamics and cellular events, for example cell death. In pharmaceutical research, the ability to quantify such metrics from high-throughput imaging systems can drive drug discovery by facilitating fast compound screening and efficacy testing. These analyses ultimately rely on robust identification and segmentation algorithms, particularly if the goal is to investigate at the level of individual cells. Many such segmentation algorithms rely on the presence of a fluorescent label. However, mounting evidence indicates fluorescent sensors can alter biological responses by effecting physiological change. Fluorescent proteins have been linked to increased cell death^1^^,^, reactive oxygen species accumulation and mitotic arrest^2^, interruption of critical cell signaling pathways^3^ and impairment of actin–myosin interactions^4^. Moreover, stable expression of fluorescent proteins requires genetic manipulation, which may not always be possible in more physiologically relevant primary cell types^5^ such as patient-derived induced pluripotent stem cells^6^. Because of this, recent years have seen renewed interest in label-free imaging approaches.

However, label-free imaging presents unique challenges. Numerous studies have developed sophisticated label-free imaging technologies, such as quantitative phase imaging^7^, but these often require users to have expertise and complex hardware. While simple brightfield and phase-contrast imaging remains the most accessible and widespread mode of label-free imaging, they offer limited contrast for resolving cells grown in a monolayer. Furthermore, the morphology of a cells in culture can vary dramatically, not only across cell types, but also due to genetics and epigenetics, microenvironmental factors, stages in the cell cycle or differentiation processes and in response to treatment, making segmentation of individual cells from label-free images of cultured cells a challenge.

Open-source and commercially available image analysis packages have been developed to tackle this problem^8–10^, but too often require careful algorithm customization and rigorous tuning of parameters specific to the cell morphology in the image. The rise in popularity of convolutional neural networks (CNNs) offers a potential solution to this problem and indeed, CNNs can learn and adapt to identify and segment objects of enormous variety. However, for a CNN to produce good results, it first requires training with high-quality datasets representative of the breadth of the problem to be solved.

In the seminal paper on U-net^11^, a CNN trained on only 35 images outperformed every other entry in the IEEE International Symposium on Biomedical Imaging 2015 cell tracking and segmentation challenge. Since U-net, there have been numerous advances applying CNNs to biological images of cells, but development of publicly available training datasets has been limited. An early open-source light microscopy dataset was DeepCell^12^, which comprised manually annotated data and trained CNN models for the single-cell segmentation of bacterial and mammalian cells. However, the DeepCell dataset consists of fewer than 50 images; arguably, this is too small to enable a trained CNN model to generalize to images beyond its training dataset. Since then, new datasets have been published, but are similarly limited in size, for example 50 images of single cells^13^, or available cell types, for example 644 images of rat CNS stem cells^14^. EVICAN, the largest such dataset so far comprises 4,600 images and 26,000 cells, including 30 different cell types and images acquired with different microscopes, modalities and magnifications^15^. Although EVICAN boasts great diversity, it averages only 5.7 cells per image, which makes it challenging to apply to all biologically relevant cell culture conditions where cell density may be substantially higher. While label-free datasets continue to be scarce, image datasets containing fluorescently labeled cells are more readily available. Moen et al.^16^ published a fluorescent imaging dataset with 63,280 annotated single cells from seven cell lines, and the Data Science Bowl 2018 featured a dataset with 37,333 manually annotated cell nuclei^17^. More recently, Stringer et al. describe the generalist segmentation algorithm CellPose trained on a dataset of approximately 70,000 segmented objects, which primarily comprised fluorescently labeled cells mixed with few light microscopy images as well as noncellular objects^18^. Although all important advances, datasets commonly used to train and benchmark CNN models in nonlive cell imaging literature are still much larger by comparison: the widely used Microsoft COCO dataset^19^ consists of 328,000 images with a total of 1.5 million segmented instances, and the Open Images V6 dataset^20^ consists of more than 900,000 images with 2.7 million segmented instances. Therefore, to maximize the potential of applying CNNs to label-free cell segmentation across all different cell morphologies, a large, high-quality dataset is crucial.

In this study, we present LIVECell (Label-free In Vitro image Examples of Cells), a new dataset of manually annotated, label-free, phase-contrast images of 2D cell culture. LIVECell consists of more than 1.6 million annotated cells of eight morphologically distinct cell types, grown from early seeding to full confluence, and has undergone rigorous quality assurance to minimize bias in the annotations. As a proof of concept of the use of LIVECell, we also present trained models developed to segment individual cells, for application in new research to enable label-free single-cell studies. Finally, in the interest in standardizing evaluation of such models, we propose a suite of benchmarks, which will readily facilitate continued development and performance comparison of future models.

## Results

### LIVECell

**Fig. 1:**
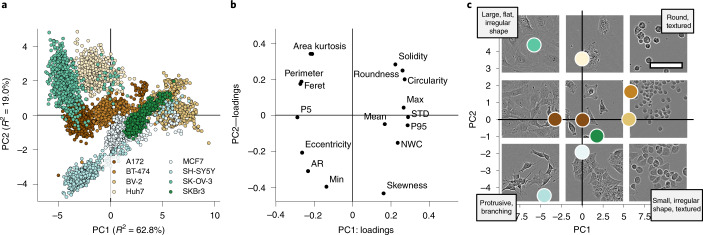
Morphological diversity of cell types comprising LIVECell visualized using PCA. **a**, Scatter plot of the first two principal components demonstrate the diverse spread of morphologies represented by images in LIVECell between and within cell types. **b**, Loading plot of PCA shows how each morphology metrics influence the directions of the component values. **c**, Representative examples of images on each axis and quadrant of the principal component plot with their principal component values plotted. Morphological interpretations, based on the loading values, are provided for each quadrant. Abbreviated metrics names are explained in the Methods. Scale bar represents 100 µm and applies to all images.

LIVECell consists of 5,239 manually annotated, expert-validated, Incucyte HD phase-contrast microscopy images with a total of 1,686,352 individual cells annotated from eight different cell types (see Supplementary Note). These cell types, spanning the small and round BV-2 to large and flat SK-OV-3 and neuronal-like SH-SY5Y, were chosen to maximize diversity to ensure LIVECell’s broad use for future machine learning development. Principal component analysis (PCA) of commonly used cell morphology metrics reveals the extent of that diversity, showing distinct clusters for each chosen cell type (Fig. 1). LIVECell also features annotated images of cells grown from the initial seeding phase to a fully confluent monolayer (Supplementary Fig. 2), resulting in great variation in cell size, that is, very small to over 6,000 µm^2^, and cell counts per image, that is, very few to over 3,000 objects (Supplementary Fig. 3). Whereas previous efforts are limited in terms of cell density^12,13,15^, LIVECell enables the training of segmentation models with applicability to the entire time course of a typical cell biology experiment.

To ensure annotation quality given such challenging images, several precautions were taken. First, the images were annotated by a dedicated team of professional annotators (CloudFactory) that received training on cell segmentation by an experienced cell biologist rather than crowdsourcing annotators. Second, images were split into balanced batches that spanned cell types and experiment time points, using a design of experiments approach^21^ and uploaded for annotation batch by batch. This batchwise approach was done to minimize the risk of introducing bias into any single part of the dataset as annotators will become more experienced and possibly more accurate as the project progresses. Last, all images passed through two rounds of quality assurance to ensure top quality, first by an annotation manager and then by an experienced cell biologist.

### LIVECell benchmark suite

We have designed a series of evaluation tasks that exploit the diversity and breadth of data available in LIVECell while also providing a platform for researchers to fully assess the suitability of their given model design for cell segmentation. Each test within this suite of benchmarks focuses on a different aspect of performance:

1. LIVECell-wide train and evaluate: here, we include all cell types in LIVECell for training and evaluate the models on the entire test dataset as well as each individual cell type. In addition to providing information on overall model performance, this task provides high-level insight into which cell types and morphological characteristics may be difficult for a model to adapt to.
2. Single cell-type train and evaluate: to allow comparison of the relative challenge imposed by the different cell types, this task trains and evaluates a model on a single cell type. This permits focused and small-scale experimentation as well as provides an opportunity for fine-tuning a model if one cell type is of particular interest.
3. Single cell-type model transferability: by training models on a single cell type and cross-evaluating it on others, this task assesses a given model’s ability to generalize to cell types unseen during training. By comparing which cell types generalize well to each other, this test provides a means to investigate how hyperparameter configuration or architecture design affects transfer learning potential.
4. Validation against fluorescence-based cell counts: this task applies trained models to an image set unseen during training containing two cell types, including one cell type that is not present in LIVECell at all, expressing a nuclear restricted red fluorescence protein. As automated cell counting based on nuclear labels is standard practice, comparing the fluorescent nuclei counts to the object count output by a trained model provides opportunity for validation and ultimately ensures biological relevancy.

To evaluate cell detection and segmentation quality in tasks 1 and 2, standard practices from the Microsoft COCO evaluation protocol^19^ were used but slightly modified to better reflect cell sizes. For our evaluation metric, we report the overall average precision (AP) and average false-negative ratio (AFNR) rather than the commonly used values at matching intersection over union (IoU) of 50%, as the overall scores provides a more extensive and rigorous assessment of model performance. Task 3 is evaluated by quantifying how well the models generalize to unseen cell types on average using a new transferability index. Task 4 is evaluating by assessing the explained variance of fluorescence-based counts compared to model-based ones and testing to how far in terms of object counts the relationship is linear.

### LIVECell benchmark performance

To serve as baseline for future method development using LIVECell, two state-of-the-art CNN-based instance segmentation models, one anchor-based and the other anchor-free, were trained and evaluated using the benchmark tasks (Fig. 3). When trained and evaluated on all of LIVECell, the two models achieve impressive segmentation results (Supplementary Fig. 4) and similar AP for segmentation (47.9 and 47.8% for LIVECell, Fig. 3a), which is comparable to each model’s published performance on Microsoft’s COCO dataset^22,23^. Further inspection of model precision across different IoU thresholds reveals disparity in performance between cell types; for example, the precision for the neuroblastoma cell line SH-SY5Y is very sensitive to the IoU threshold, whereas the breast cancer cell line SkBr3 demonstrates robust precision for all IoU levels less than 80% (Supplementary Fig. 5a,b). While precision appears similar between the two models, the anchor-based model achieved a lower AFNR than the anchor-free model (45.3 and 52.2%, respectively, Fig. 3b and Supplementary Fig. 5c,d), underscoring the importance of considering both metrics for comprehensive evaluation.

Training and evaluating on a single cell type further highlighted how the cell types represented in LIVECell vary greatly in terms of difficulty. For instance, model performance on SkBr3 cells scored quite high (detection and segmentation AP 64–66%) whereas SH-SY5Y scored much lower (AP 22–28%) relative to other cell types (Fig. 3c and Supplementary Fig. 6). Both models achieved similar AP for each of the eight cell types. Notably, both models perform better on each individual cell-type test set when trained on all cell types compared to training on that single cell type (compare Fig. 3a and c, details in Supplementary Table 7) indicating that a cell-type universal model is preferable to a specific one. The anchor-free model benefited more from training on all of LIVECell compared to the anchor-based model and increased 6.6 AP points on average compared to 2.0 (*P* = 0.01, paired *t*-test of null hypothesis that the anchor-free increase is less than or equal to that of the anchor-based). In the transferability task, models trained on a single cell type vary greatly in their ability to generalize to other cell types (Fig. 3e,f). For example, models trained on A172, BT-474, SkBr3 or SK-OV-3 perform relatively well when applied to all other cell types, achieving an average transferred AP of 28–36%; however, the opposite is observed for models trained on only BV-2 images, where we observe an average transferred AP of only 10.1 and 12.0% for the anchor-based and anchor-free models, respectively. To quantify overall transferability, we designed a transferability index, where a perfect score of 0 indicates a model on average performs just as well on unseen cell types as the cell type it was trained on with higher scores are indicative of less transferability. Using this metric, the anchor-based model better generalizes to unseen cell types overall than the anchor-free model, achieving a transferability index of 0.98 compared to 1.21.

For the final task, models trained on LIVECell were validated using an unseen image set of A172 and A549 cells expressing a nuclear restricted red fluorescence protein and nuclei were counted using commercially available software. Cells were seeded at various densities and grown past full confluence (Fig. 4, A172 in Supplementary Video 1, A549 in Supplementary Video 2 and Supplementary Fig. 7). Predicted cell counts from the anchor-free model follow nuclei counts closely over time (Fig. 4a,e) with 98 and 94% linear correlation for A549 and A172 up to 95% confluency (Fig. 4b,f). The anchor-based model performs similarly below 95% confluency (linear correlation 99% for A549 and 98% for A172). While both models display less reliable object counts in overconfluent images, the accuracy of object counts from the anchor-based model drastically deteriorates when the number of cells per image surpasses 1,300–1,500 (Fig. 4c,d,g,h). To quantify this, we performed iterative goodness-of-fit tests to evaluate the linear relationship of fluorescent-based versus model-based object counts to identify an object density threshold where each model begins to fail. Here, we observe that linearity holds for the anchor-free model at higher object counts (up to 2,031 and 1,948 objects per image for A549 and A172, respectively) compared to the anchor-based model (up to 1,403 and 1,328). To confirm accuracy of our fluorescence-based cell counts, we quantified rates of unlabeled and multinucleated cells and determined that they do not bias the results reported above (Supplementary Note).

### LIVECell scale experiments

The size of LIVECell permits investigation into how the number of instances in the training set affects segmentation performance. Anchor-free and anchor-based models were trained on subsets of the full LIVECell training set, evenly selected across cell types and time points, and evaluated on the complete LIVECell-wide test set. This revealed that the segmentation AP monotonically increased with training set size without either model reaching a saturation point (Fig. 5a–c) and false-negative ratio decreased monotonically (Fig. 5d–f), suggesting larger training sets can further improve performance both in terms of AP and AFNR. Notably, AP increases considerably when training on more than 2 and 5% of LIVECell (that is, 24,197 and 51,488 instances, comparable to the number of annotated objects in the largest pertinent datasets so far^15,18^). Overall, increasing the training set size from 2 to 100% of LIVECell results in a 7.7 and 11.5 point increase in AP for the anchor-based and anchor-free models, respectively. It is also noted that while both models achieve similar performance when trained on all LIVECell (47.9 and 47.8% AP), the anchor-based model performs better on smaller subsets (for example, 40.2% AP at 2% of LIVECell, compared to 36.2%).

### Assessing transferability between LIVECell and other datasets

Although LIVECell is a highly comprehensive dataset for cell segmentation, it does not fully cover all aspects of biology and imaging. Due to equipment availability, all LIVECell images were acquired using the same imaging platform and magnification in contrast to multi-instrument datasets such as EVICAN^15^ and CellPose^18^. To demonstrate that LIVECell is a valuable resource for light microscopy imaging modalities and magnifications beyond our imaging platform, we applied models trained on LIVECell to the EVICAN and CellPose evaluation datasets, which includes Zernike phase-contrast, fluorescence and brightfield images from multiple instruments and multiple magnifications. We found that LIVECell-trained models transfer out-of-the-box given appropriate digital preprocessing (Supplementary Note). In fact, with no additional training on data beyond LIVECell, our anchor-free and -based models achieve an overall AP of 36.7 and 59.6% on the EVICAN easy evaluation dataset, outperforming the previously reported EVICAN results of 24.6% (Supplementary Table 4). Furthermore, our models achieve similar accuracy to the CellPose baseline models on the CellPose evaluation dataset (AP = 24.5 and 26.9%, Supplementary Fig. 13). The CellPose results were particularly surprising given its dataset mostly comprises fluorescence-based images. In contrast, we find that the CellPose generalist model struggles to segment certain cell types and many of the highly confluent LIVECell evaluation images (AP = 13.9%, Supplementary Note). All of this evidence taken together highlights how such a large, high-quality dataset such as LIVECell fills a critical need in the field.

## Discussion

**Fig. 2:**
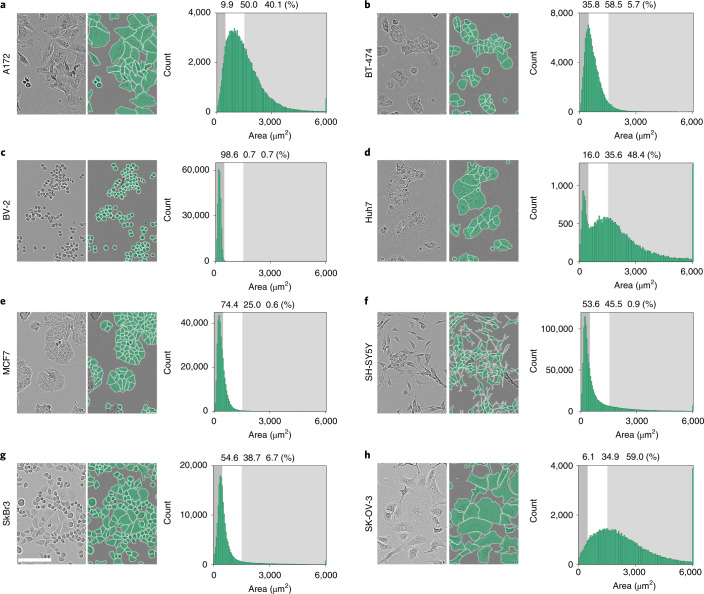
Illustrative examples of annotated phase-contrast microscopy images and histograms showing cell size distributions of all cell types in LIVECell. Example images for **a**, A172, **b**, BT-474, **c**, BV-2, **d**, Huh7, **e**, MCF7, **f**, SH-SY5Y, **g**, SkBr3 and **h**, SK-OV-3 cells are shown in pairs, with the original phase-contrast image on the left and the overlaid annotations shown on the right in green. Images demonstrate morphological variety represented by the chosen cell types. Histograms show cell size distributions in µm^2^ for each cell type. On each histogram, the vertical color panes indicate the different cell size categories used for model evaluation and the percentages above each pane indicate how many in each cell type belong to each size category. The left-hand gray pane indicates small cells (defined as smaller than 320 µm^2^), the middle white pane indicates medium-sized cells (between 320 and 970 µm^2^) and the right-hand gray pane indicates large cells (larger than 970 µm^2^). Scale bar represents 150 µm and applies to all images.

Achieving accurate object-by-object segmentation is a challenging task in any machine learning application and while fully unsupervised approaches are being developed, current CNN-based instance segmentation typically require large, annotated datasets and well-designed benchmarks to fairly assess performance and bias. LIVECell introduces the largest high-quality resource for label-free cell segmentation. By including a wide variety of cell morphologies (Fig. 1) and confluence levels (Supplementary Fig. 2), LIVECell can facilitate development and assessment of segmentation algorithms for biologically relevant cell culture experiments. In contrast to other instance segmentation datasets, LIVECell also presents challenges unique to label-free 2D cell culture image data. First, the average number of objects per image in LIVECell is 313 (Supplementary Fig. 3), which is substantially higher than typical instance segmentation datasets such Microsoft COCO^19^ (7.8 objects per image) or dense datasets such as SKU-110K^24^ (147.4 objects per image). To avoid slow performance speed and heavy memory requirements, an optimal CNN model for LIVECell must be appropriately designed to handle these high object counts. The two models presented here demonstrate linear increases in processing time with object count (see details on evaluation in Supplementary Note) and we propose that an ideal model design would minimize or bypass this linear trend. Furthermore, we found the definitions for small, medium and large object size categories in the standard COCO evaluation protocol^19^ did not appropriately reflect cell sizes observed in LIVECell (Fig. 2) and biased size-based evaluations, which is exacerbated by the nonuniform distribution of object sizes within LIVECell (Supplementary Fig. 3). Only by adjusting the size category definitions to better reflect the biology present were we able to separately evaluate segmentation accuracy on small, medium and large objects (Supplementary Table 7).

**Fig. 3:**
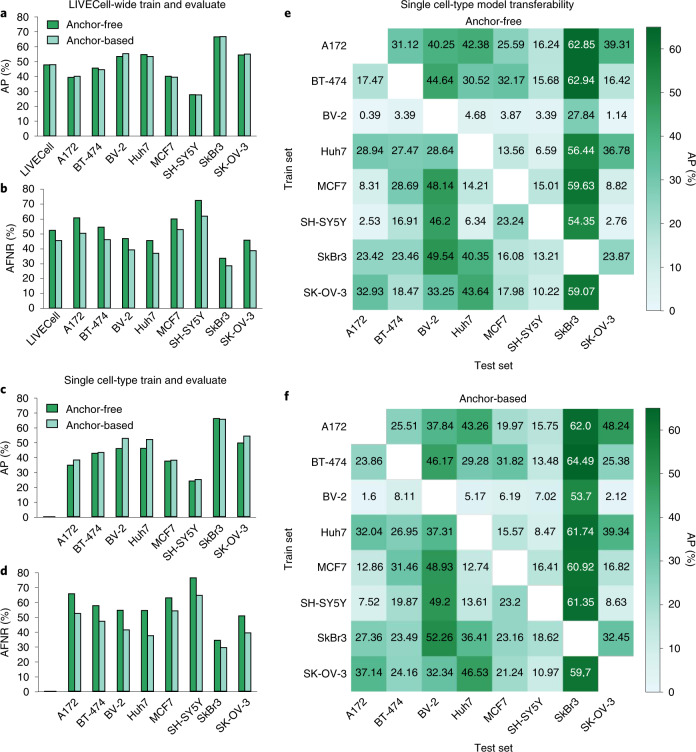
Performance evaluation of CNN models trained on LIVECell. **a**–**f**, Bar charts of cell segmentation performance, as reported by mask AP (%), for the LIVECell-wide train and evaluate task (**a**) and single cell-type train and evaluate task (**c**), cell detection performance, as reported by mask AFNR (%) for the LIVECell-wide train and evaluate task (**b**) and single cell-type train and evaluate task (**d**), as well as heatmaps for all possible transfers on the single cell-type model transferability test for the anchor-free (**e**) and anchor-based model (**f**) as reported by AP.

The anchor-based and anchor-free segmentation models we trained with LIVECell show convincing segmentation performance (Fig. 3) on par with their published performance with Microsoft’s COCO dataset^22,23^. Comparing our models to those trained on similar datasets, such as EVICAN, we observe substantially higher segmentation accuracy, where our models achieve AP scores greater than 80% using an IoU threshold of 50% (Supplementary Table 7 and Supplementary Figs. 5 and 6) compared to the 61% reported in the EVICAN study, highlighting the benefit of training on a larger-scale dataset^15^. Certain cell types proved to be particularly difficult for the segmentation models. For example, the accuracy scores for the neuroblastoma cell line SH-SY5Y appears notably lower than that of the other cell types. Indeed, neuronal cells have a unique morphology compared to the other cell types, tending to be highly asymmetric and concave-shaped due to their characteristic branching neurites. Asymmetric and concave morphologies have proved challenging for cell segmentation models^18^ and put high demands on models to learn long-range dependencies to correctly assign pixels to the correct object instance. Convolutions, the cornerstone of CNNs, effectively describe translation equivariance and locality but struggle to model long-range dependencies. Recent model architectures^25,26^ aim to relieve these limitations and may be necessary to accurately segment this type of specific morphology.

**Fig. 4:**
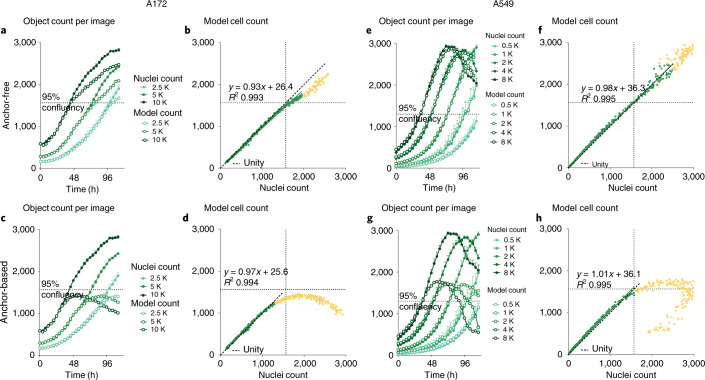
Validation of anchor-free and anchor-based model using fluorescent nuclei count. **a**–**h**, Predicted model counts are compared to fluorescence nuclei counts on A172 and A549 cells. Time course graphs show per-image object counts across different cell seeding densities over time for fluorescent nuclei and the models for the anchor-free (**a**) and anchor-based (**c**) model for A172 cells and the anchor-free (**e**) and anchor-based (**g**) model on A549 cells. Correlation plots for each image show *R*^2^ > 0.99 with a gradient close to 1 when comparing nuclei count and label-free predictions of the anchor-free (**b**) and anchor-based (**d**) models for A172 cells and anchor-free (**f**) and anchor-based (**h**) models for A549 cells. The yellow markers highlight data removed from the correlation calculations. On all graphs, the dotted line represents the 95% cell confluence level. Data are shown as mean ± s.e.m. for *n* = 4 images (**a**,**c**) and *n* = 3 images (**e**,**g**).

**Fig. 5:**
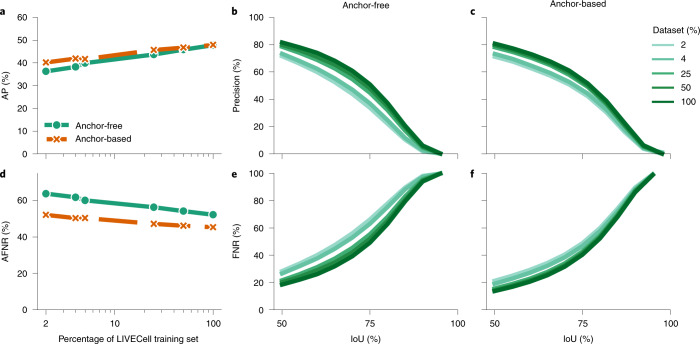
Impact of scale of dataset on segmentation performance. Each model was trained on subsets of the LIVECell training set, corresponding to 2, 4, 5, 25, 50 and 100% of total number of images. **a**,**d**, The resulting models were then evaluated by calculating segmentation AP on the complete LIVECell test (**a**) and AFNR (**d**). To further explore the effects of increasing the dataset size we broke down the metrics to each IoU level between 50 and 95% with a step size of 5%. **b**,**c**,**e**,**f**, The precision per IoU for the anchor-free (**b**) and anchor-based (**c**) models trained on different amounts of the dataset was calculated, as well as the FNR for the same anchor-free (**e**) and anchor-based models (**f**).

Beyond direct application of LIVECell-trained models, LIVECell also offers a robust dataset for pretraining before fine-tuning on small datasets from other instruments due to its size, morphological diversity and high object density. In particular, evaluating the extent to which models trained on LIVECell’s Incucyte HD phase-contrast images benefits the analysis of other light microscopic modalities, including brightfield and differential interference contrast images, and offers an interesting domain transfer problem for future research. In terms of biological limitations, many cell types can become overconfluent, continuing to divide as cells pack closer and closer together. To explore how our models perform in such a scenario, we compared model-predicted cell counts to fluorescence-based cell counts on images of cells growing overconfluent until they started to die off (Fig. 4, Supplementary Videos 1 and 2 and Supplementary Fig. 7). The anchor-free model was able to generalize well to overconfluent images without adjustments, although it slightly underreported A172 cell counts and showed greater variance of A549 cell counts in overconfluent images. On the other hand, the anchor-based model struggled when cells reached high confluence and began making erratic predictions, which is particularly intriguing considering that the anchor-based model showed a lower false-negative ratio on the LIVECell segmentation tasks (Fig. 5d–f). Therefore, while both models demonstrate similar detection and segmentation performance on LIVECell, they differ greatly in their ability to extrapolate to more extreme experimental conditions.

This observed performance difference between the anchor-based and anchor-free models on highly confluent images may be a result of their inherently different detection mechanisms. Anchor-based detection means that the localization of objects is based on a set predefined anchor boxes, that is rectangles of different sizes and aspect ratios. These anchor boxes are used to predict the existence of objects at each spatial location in the CNNs intermediate representation of the original image. The most confident top few anchor boxes over a certain threshold are selected to represent the bounding boxes of predicted objects. Then for instance segmentation, the area within each bounding box is segmented to outline the contained object as well. Anchor-free detection on the other hand, means that the object detection does not depend on a predefined set of anchor boxes. Instead, it uses a fully convolutional one-stage (FCOS) object detection^27^ to predict the center points of objects and directly define bounding boxes, circumventing the need for additional hyperparameters. We speculate that these differences may be due to the requirement to tune anchor box sizes for the LIVECell segmentation benchmarks may cause the model to struggle at detecting cells at even higher density. Fine-tuning the anchor boxes for images with higher confluence may mitigate the performance difference but could sacrifice model accuracy at lower densities. These observations open possibilities for future research into the exact cause of the performance discrepancy and how different CNN architectures handle overconfluent images.

It should be noted that when setting out to annotate LIVECell, we intentionally chose not to attempt separation of a large cell mass into individual cells. Annotations in such regions where cell boundaries are not readily visible (arrows, Supplementary Fig. 2) would be arbitrary and risk introduction of bias, which is consistent with EVICAN^15^ that also uses partially annotated images. We do not view this as a limitation, but rather believe that this choice will improve the applicability of models trained on LIVECell. In most applications, segmentation quality is more important than detecting every possible object if detection is not biased, for example measures of morphology statistics or within-cell fluorescence when coupled with fluorescent imaging. Poor segmentation will degrade data quality, which will propagate through downstream use of segmentation results. Even worse, if segmentation performance is biased, this bias may compromise any biological findings relying on segmentation. We note, however, that LIVECell’s partially annotated images may introduce a risk that models struggle to train well and miss more cells than necessary. However, we do not experience any such problems and all models converge well (Supplementary Figs. 9 and 10) and the dataset scale experiments shows that not only does the precision increase with the increase scale, but the false-negative ratio also improves (Fig. 5d–f). Furthermore, both models transfer well to other imaging platforms out-of-the-box (Supplementary Fig. 12). The size and diversity of LIVECell and its rigorous benchmarks allows quantification of this bias for the first both across different cell morphologies and different levels of confluency.

Two-dimensional light microscopy is an accessible source of cellular imaging material that allows high throughput. This modality enables massive amounts of image material to be collected noninvasively to represent datasets containing millions of cells, which in turn facilitates biological phenomena to be studied with great statistical power. To be able to compensate for lack of imaging resolution, sophisticated imaging processing pipelines are necessary to capture subtle changes in biology. Accurate cell-by-cell segmentations open the possibilities to explore more complex biological questions, for example tracking subpopulation response to a treatment condition, investigating migration dynamics in a segmented time lapse. With LIVECell to enable CNN-model development for 2D cell culture images, we envision such models will serve as the basis for analyses pipelines that target such exciting and physiologically relevant topics in biology and medicine.

## Methods

### Data collection and annotation

#### Image acquisition

To ensure the dataset covered a wide variety of cell morphologies, a diverse set of eight cell types were chosen (A172, BT-474, BV-2, Huh7, MCF7, SH-SY5Y, SkBr3 and SK-OV-3; see summary of cell type and morphology in Table 1). All cell lines were purchased from ATCC, except Huh7 (CLS cell line services) and BV-2 (Interlab Cell Line Collection) and were cultured as per suppliers’ recommendations. Several wells for each cell type were seeded in 96-well plates (Corning) and imaged over the course of 3–5 d, every 4 h using an Incucyte S3 Live-Cell Analysis system (Sartorius) equipped with its standard CMOS camera (Basler acA1920-155um). The Incucyte HD phase-contrast imaging algorithm allows visualization of phase delays produced by cells without the phase annulus or phase ring found in conventional Zernike phase images. Because of this, LIVECell phase-contrast images are characterized by less pronounced halo artifacts and more high-frequency content than other phase-contrast modalities. Phase-contrast images were acquired using a ×10 objective from two positions in each well adding up to a total of 1,310 images (1,408 × 1,040 pixels corresponding to 1.75 × 1.29 mm^2^) that were each cropped into four equally sized images (704 × 520 pixels corresponding to 0.875 × 0.645 mm^2^) that were then annotated.

**Table 1:** Overview of cell lines used to construct the LIVECell dataset

Cell type	Species	Type	Wells	Time length	Why chosen
A172	Human	Glioblastoma	4	3 d	General adherent cell morphology. Over grow each other at higher densities.
BT-474	Human	Breast cancer	4	5 d	Grow in rafts. Challenging to locate cell boundaries clearly.
BV-2	Mouse	Microglia	4	3 d	Small spherical morphology. Homogeneous population.
Huh7	Human	Hepatocellular	3	4 d	Low contrast cells. Challenging to locate cell boundaries clearly.
MCF7	Human	Breast cancer	4	3 d, 16 h	Grow in rafts. Challenging to locate cell boundaries clearly.
SH-SY5Y	Human	Neuroblastoma	4	3 d, 12 h	Neuronal morphology with long protrusions. Overlapping cells.
SkBr3	Human	Ovarian cancer	4	3 d, 12 h	Low contrast cells. Heterogeneous morphologies.
SK-OV-3	Human	Breast cancer	4	3 d	Heterogeneous morphology.

#### Image annotation

Since image annotation is labor-intensive, a managed team of professional annotators (CloudFactory) were trained to perform single-cell segmentation. To reduce the risk of introducing bias due to annotators gaining experience over the course of the task, the dataset was split into eight batches balanced over cell types, timestamps and wells using generalized subset designs^21^ as implemented in MODDE v.12.1 (Sartorius Data Analytics). The dataset was then uploaded for annotation batch by batch. Before starting any annotation, one well per cell type was selected at random to be included in a test dataset for machine learning model evaluation. Selecting a complete well, instead of the standard practice of selecting images at random, ensure that the training and test set are physically separate, greatly reducing the risk of data leakage by, for example, the plate texture.

All cells in all images that could be unambiguously identified by an experienced cell biologist were then annotated by outlining them with polygons using a commercially available, cloud-based, annotation software (V7Labs Darwin). To train the annotation team, the annotation managers were first trained on a one-to-one basis until they were able to annotate the images with sufficient quality. To scale up, the annotation managers then trained the remaining team members.

#### Quality assurance

Due to the low contrast and high object density making annotation challenging, two levels of quality assurance were used to minimize the risk of introducing label noise. The first level was performed by the annotation managers and second round by an experienced cell biologist. During quality assurance, each image was inspected and any images with faulty annotations were sent back to the annotators along with feedback. Examples of faulty annotations include cells that were not annotated but should have been, cells outlined with a too course polygon leading to a jagged segmentation mask, large cells that had been split into multiple cells, debris annotated as cells and so on. An image sent back to the annotator was revised before sent for new quality assurance. If the annotation manager approved the image, it was sent to a cell biologist for final approval. Images passing both rounds of approval were included in LIVECell. To assure that the annotation managers assessments stayed consistent, there were frequent follow-up calls where the cell biologist provided feedback on difficult cases directly to the annotation managers.

#### Fluorescence-based cell counts

To enable validation of cell detection by LIVECell-trained models in biologically relevant cell culture conditions, a separate set of A172 and A549 cells expressing a nuclear restricted red fluorescence protein were seeded at various densities and cultured for 5 d. Phase-contrast and red fluorescent images were captured at ×10 magnification using an Incucyte S3 Live-Cell Analysis system. Fluorescence-based cell counts were measured by detecting and segmenting the fluorescently labeled nuclei using the commercially available Incucyte Basic Analyzer. These counts were then used to evaluate the cell counts obtained label-free using segmentation models trained on LIVECell.

### Data exploration

#### Multivariate data analysis of cell morphology

To measure the morphology of cells, a set of 17 metrics was chosen and measured for each individual cell in LIVECell. These metrics were chosen to represent different cell features, including size (area, perimeter, Feret’s diameter), phase-contrast intensity (mean; minimum; maximum; P5, fifth percentile; P95, 95th percentile) and intensity distribution (skewness, kurtosis, normalized weighted centroid (NWC)), texture (standard deviation of intensity (STD)), and shape (eccentricity, roundness, circularity, solidity and aspect ratio (AR)). Image annotations were used to create a region of interest for each cell using OpenCV v.4.4.0.46, and standard metric calculations were run using scikit-image v.0.17.2 or SciPy v.1.5.2.

To provide an overview of the cell diversity represented by LIVECell we applied multivariate data analysis, specifically PCA. The morphology metrics were preprocessed based on their distributions over all cells in LIVECell. The image-wise morphology metric averages were then calculated by summing the metrics over all cells in each image and dividing by the number of cells per image. The image-wise averages were then standard-scaled, that is, mean-centered to zero and scaled to unit-variance. Last, a two-component PCA-model was fitted and visualized using scatterplots of PCA scores over image-wise average and PCA loading scatterplots showing the morphology metrics influence on the PCA-component directions. PCA was calculated using Python libraries scikit-learn v.0.22.2 and visualized using matplotlib v.3.1.1.

### Training segmentation models

#### Instance segmentation model architectures

Two state-of-the-art CNN-based instance segmentation models were trained on LIVECell to evaluate the benchmark tasks’ difficulty. The two models used inherently different object detection mechanisms: anchor-based and anchor-free. The anchor-based model was an adapted version of Cascade Mask RCNN^28^ using a ResNest-200 backbone^23,29^. The anchor-free model was based on CenterMask^22,30^ an anchor-free one-stage architecture with a VoVNet2-FPN backbone using FCOS detection^27^. Nine models of each architecture were trained on LIVECell, one model on the whole dataset (LIVECell-wide train and evaluate benchmark) and one for each of the eight cell types (single cell-type train and evaluate benchmark).

#### Model training

All training used transfer learning by starting with weights pretrained on the MS-COCO 2017 dataset^19^ that were then fine-tuned on LIVECell. Training used a stochastic gradient descent-based solver, a batch size of 16 images per iteration (distributed to two per graphical processing unit (GPU)), a momentum of 0.9, a linear learning rate warmup with warmup factor of 0.001 and other training parameters listed in. All models were trained on a NVIDIA DGX-1 server hosting eight NVIDIA Tesla V100 GPUs with 32 Gb of GPU RAM each, dual 20-Core 2.2 GHz Intel Xeon CPUs and 512 Gb system RAM. The anchor-based model was implemented using the Python programming language v.3.6.10 (Python Software Foundation, https://www.python.org/), the deep learning framework PyTorch^31^ v.1.5.0 and the object detection library Detectron2 (ref. ^32^) v.2.1. The anchor-free model was implemented using Python programming language v.3.7.7, PyTorch v.1.5.0 and Detectron2 v.0.3.

All training was run for a predefined set of iterations and the loss on a validation set, separate from the training and test sets, were monitored to assess model over- and under-fitting. Model checkpoints were saved and used for evaluation based on which had the lowest validation loss (Supplementary Figs. 9 and 10) on the rationale that the lowest validation loss represents a good balance between an under- and over-fitted model. For the anchor-free model trained on SH-SY5Y the warmup iterations were increased from the default 1,000 to 5,000 as the default parameters resulted in unstable gradients due to the learning rate increasing too quickly. Because the model did not properly converge in 10,000 iterations, we extended that training to 20,000 iterations in total.

For normalization, pixel intensity values were centered around zero by subtracting by the global average pixel value for the dataset (128), and then divided by the global standard deviation (11.58). To reduce the risk of overfitting, all training used multi-scale data augmentation meaning that image sizes were randomly changed from the original 520 × 704 pixels to a size with the same ratios, but shortest side set to one of (440, 480, 520, 580, 620) pixels.

During early experiments, the anchor-based model struggled to detect small cells (for example, BV-2, Fig. 2c) using parameters from the original implementation^23^. To better segment these cells, the predefined anchor box sizes were reduced to (4, 9, 17, 31, 64, 127 pixels) compared to standard (32, 64, 128, 256, 512 pixels) and anchor generator aspect ratios are changed to (0.25, 0.5, 1, 2, 4) compared to the standard (0.5, 1, 2 pixels).

### Benchmarking

#### Segmentation benchmarks

To evaluate models’ performance on all benchmarks, a slightly modified version of the COCO evaluation protocol^19,33^ was used. The COCO evaluation protocol is widely used in machine learning to evaluate performance of how well objects are detected and segmented compared to ground truth annotations, and report overall AP of detection at a certain degree of overlap between the prediction and ground truth. This is calculated in several steps. First, the degree of overlap between each prediction and its closest ground truth object is quantified using the IoU given by:

$${\mathrm{IoU}} = \frac{{\rm{Pred}} \cap {\mathrm{Target}}}{{\rm{Pred}} \cup {\mathrm{Target}}}$$

If the IoU between the prediction and the closest ground truth target is larger than a certain threshold, the ground truth target is deemed as correctly detected. For all ground truth objects, the detection performance is then quantified using the precision and recall metrics given by:

$${\mathrm{Precision}} = \frac{{{\mathrm{TP}}}}{{{{{\mathrm{TP}}}} + {\mathrm{FP}}}}$$

$${\mathrm{Recall}} = \frac{{{\mathrm{TP}}}}{{{{{\mathrm{TP}}}} + {{{\mathrm{FN}}}}}}$$

where TP is the number of true positives, FP is number of false positives and FN is number of false negatives. The recall is monotonically increasing with the IoU threshold but the precision, *p*, is recalculated to be monotonically decreasing by interpolating the precision at multiple recall levels by:

$$p_{\mathrm{{interp}}}\left( r \right) = \mathop {{\max }}\limits_{r{\prime} \ge r} p(r{\prime})$$

where $p(r{\prime})$ is the precision given a recall value *r*, *r’* is each recall value greater or equal to *r* at the IoU threshold. The AP at the IoU threshold, AP_IoU_, is then given by the area under the curve when plotting the precision against recall for the instance predictions given at the IoU threshold given by:

$${\mathrm{AP}}_{\mathrm{{IoU}}} = \mathop {\sum }\limits_{i = 1}^{n - 1} (r_{i + 1} - r_i)p_{\mathrm{{interp}}}(r_{i + 1})$$

where *n* is the number of instance predictions. To evaluate LIVECell segmentation benchmarks, the COCO-standard overall AP is used, meaning that the average AP over IoU thresholds from 0.5 to 0.95 with a step size of 0.05 is used instead of a single IoU threshold. In mathematical notation:

$${\mathrm{AP}} = \frac{{{\mathrm{AP}}_{0.50} + {\mathrm{AP}}_{0.55} + \ldots + {\mathrm{AP}}_{0.95}}}{{10}}$$

The overall AP is far more conservative than AP_0.50_ commonly used in literature^15^, which allows for 50% segmentation mismatch to classify a detection as correct.

To allow fine-grained evaluation of performance depending on object size, AP is also calculated separately for objects divided into different size categories. To better reflect sizes of cells, custom threshold sizes for small, medium and large cells are used (see distributions and thresholds in Fig. 2). Namely, small cells are set as smaller than 320 µm^2^ (corresponding to 500 pixels), medium cells between 320 and 970 µm^2^ (correspond to 1,500 pixels) and subsequently large cells as larger than 970 µm^2^. Because there are so many instances per image in LIVECell, the maximum detections per image was increased to 3,000 compared to the standard 100.

Precision and AP quantify how correct detected instances are but provide little insight into the accuracy of the number of detected instances. We use the false-negative ratio (FNR) to quantify detection performance, where the false-negative ratio at certain IoU threshold is given by:

${\mathrm{FNR}}_{{\mathrm{IoU}}} = 1 - {\mathrm{Recall}}_{{\mathrm{IoU}}}$ where ${\mathrm{Recall}}_{{\mathrm{IoU}}}$ is the recall of the predictions at the IoU threshold. Analogous to AP, we calculate the AFNR over multiple IoU thresholds as:

$${\mathrm{AFNR}} = \frac{{{\mathrm{FNR}}_{0.50} + {\mathrm{FNR}}_{0.55} + \ldots + {\mathrm{FNR}}_{0.95}}}{{10}}$$

to quantify the overall detection performance.

#### Cell-type generalization benchmark

The single cell-type model transferability benchmark enables quantification of how well models can generalize to unseen cell types, by training a single LIVECell cell type and evaluate on the remaining ones. We report the generalization performance by comparing the model performance on the cell type it was trained on to its performance on the other cell types. Since cell types vary in general difficulty, the log-transformed ratio is used for comparison instead of the absolute performance differences. For each cell type, the relative generalization performance on one cell type is given by the log_2_-transformed ratio of the training set cell type compared to evaluation cell type. In mathematical notation, the generalization performance of model trained on cell type A evaluated on cell type B is given by: $r_{{\mathrm{A,B}}} = \log _2\frac{{{\mathrm{AP}}_{\mathrm{B}}}}{{{\mathrm{AP}}_{\mathrm{A}}}}$ where AP_A_ denotes the AP for cell type A. The transferability index is then given by the negative mean log_2_-ratio:

$${{{\mathrm{transferability}}}}\;{{{\mathrm{index}}}} = - \frac{1}{{n (n - 1)}}\mathop {\sum }\limits_{c_1 \in C} \mathop {\sum }\limits_{c_2 \in C,c_1 \ne c_2} r_{c_1,c_2}$$

where *n* is the number of cell types and *C* the set of available cell types. The rationale for negating the mean is that in most cases the transferred performance is lower than for the cell type used for training, resulting in negative score. A positive score, where lower is closer in performance, is arguably more intuitive to interpret than a negative unbounded number. The cell-type generalization benchmark was implemented in Python v.3.6.7 and Pandas v.1.1.5.

### Validation against fluorescence-based cell counts evaluation

To quantify how well models can extrapolate in terms of cell counting compared to fluorescence-based cell counts, we use two metrics based on linear regression. First, we test how much variance in fluorescence-based cell counts is explained by the model cell counts in images with fewer than 1,600 objects, roughly corresponding to 95% confluence and the confluency level covered by LIVECell. In mathematical notation, the variance explained, *R*^2^, is given by:

$$R^2 = 1 - \frac{{{\mathrm{SS}}_{\mathrm{{residual}}}}}{{{\mathrm{SS}}_{{\mathrm{total}}}}}$$

Given fluorescent-based cell counts $y_{\mathrm{{fluorescence}}}$ and model-based cell counts $y_{{\mathrm{model}}}$, the residual sum of squares, SS, of images $i$ is given by:

$${\mathrm{SS}}_{\mathrm{{residual}}} = \mathop {\sum }\limits_i \left( {y_{{\mathrm{fluorescence}},i} - y_{{\mathrm{model}},i}} \right)^2$$

And given the average fluorescent cell count $\hat y_{\mathrm{{fluorescent}}}$, the total sum of squares is given by:

$${\mathrm{SS}}_{{\mathrm{total}}} = \mathop {\sum }\limits_i \left( {y_{{\mathrm{fluorescence}},i} - \hat y_{{\mathrm{fluorescent}}}} \right)^2$$

Second, we quantify for how many objects the models are able to sustain the linear relationship by testing goodness-of fit of linear regression at increasing fluorescent-based counts of objects. The goodness-of fit-test fails when a nonlinear regression model explains a statistically significantly larger degree of the variation in fluorescent-based cell counts compared to the linear one. For a given maximum fluorescence-based count of objects, we fit a linear regression model of the fluorescent-based counts from all images with less than or equal to the maximum number of cells using the model-based counts as regressor. Then we fit a nonlinear model using the same response and regressor, more specifically K-nearest neighbor regression using five neighbors (as implemented in Scikit-learn v.0.24.1). To test goodness-of-fit of the linear model, we test the null hypothesis that the residuals of the linear and the nonlinear model have equal variances according to Levene’s test^34^ (Scipy v.1.3.1). The test is then repeated while incrementing the maximum fluorescence-based count per image until the null hypothesis is rejected using $P = 10^{ - 5}$ as threshold. The threshold is selected to correspond to when the model predictions break down qualitatively. The maximum number of objects where the null hypothesis is first rejected is then reported as the limit of the model’s linear extrapolation.

#### Dataset size experiments

To further explore the impact of dataset size on model performance the dataset was divided into subsets corresponding to 2, 4, 5, 25, 50 and 100% of the total dataset size. The subsets were created by taking every *n*th image from the training dataset *n* $\in \{ 50,25,20,4,2,1\}$. To compare to existing datasets, 2% of LIVECell roughly corresponds to the number of instances in EVICAN^15^ and 5% to CellPose^18^. Both anchor-based and anchor-free models were trained on these subsets and then evaluated against the full LIVECell test dataset. For each subset and model, models were checkpointed during training and the checkpoints where validation loss stopped decreasing was selected to prevent overfitting.

### Reporting Summary

Further information on research design is available in the Nature Research Reporting Summary linked to this article.

## Online content

Any methods, additional references, Nature Research reporting summaries, source data, extended data, supplementary information, acknowledgements, peer review information; details of author contributions and competing interests; and statements of data and code availability are available at 10.1038/s41592-021-01249-6.

## Supplementary information

Supplementary Information Supplementary Notes, Figs. 1–14 and Tables 1–4.

Reporting Summary

Supplementary Table 1 List of images with duplicate annotations.

Supplementary Table 2 List of images annotated twice.

Supplementary Table 3 Detailed annotation performance on LIVECell benchmarks.

Supplementary Table 4 Detailed annotation performance on LIVECell scale experiments.

Supplementary Video 1 Qualitative segmentation performance on A172 cells.

Supplementary Video 2 Qualitative segmentation performance on A549 cells.

## Data Availability

The LIVECell dataset, trained models and config files to apply the models have been deposited at https://sartorius-research.github.io/LIVECell/ and 10.6084/m9.figshare.14931555.

## Code availability

All software source code is available at https://github.com/sartorius-research/LIVECell.
