# Supplementary material for: LIVECell—A large-scale dataset for label-free live cell segmentation
Source: Nat Methods. 2021 Aug 30;18(9):1038–45. doi: 10.1038/s41592-021-01249-6 (PMC8440198; doi:10.1038/s41592-021-01249-6)
Supplement: Supplementary file 1 — Supplementary Notes, Figs. 1–14 and Tables 1–4. [file 41592_2021_1249_MOESM1_ESM.pdf]

nature portfolio

https://doi.org/10.1038/s41592-021-01249-6

Supplementary information

LIVECell—A large-scale dataset for label-free live cell segmentation

In the format provided by the authors and unedited

Supplementary Note: Detailed dataset description

LIVECell consists of 5 239 manually annotated phase contrast microscopy images with a total of 1 686 352 annotated cells (see details in Supplementary Table 1). Due to software issues in the beginning of the annotation project, a limited number of images were annotated twice adding up to 5 383 sets of annotations (Supplementary Table 2, see list of duplicate images in Supplementary Table 5). Additionally, 143 images contain cells that have been annotated twice (see list of annotation files in Supplementary Table 6). Despite that special care was taken to reduce bias, there are more annotated cells of small cell types compared to larger ones (Supplementary Figure 3a). For instance, there are more than 200 000 training set cells of small and globular BV-2 cells compared to roughly 20 000 of large and flat Huh-7. Cells growing in tightly packed rafts, such as MCF7, are also overrepresented in LIVECell due to a larger number of cells per image.

Certain cell types in LIVECell, predominately BV-2, BT-474, Huh7 and SH-SY5Y, form densely packed clusters where it is not possible even for an experienced cell biologist to detect boundaries between individual cells (see arrows, Supplementary Figure 1). Since LIVECell provides benchmarks for identification of individual cells, clusters were actively excluded from annotation based on the rationale that it is not possible to properly validate segmentation quality in these regions. In previous works, where a much smaller number of cells at lower degree of confluence have been annotated, these types of clusters have not been present. But due to the diversity of cell sizes (Supplementary Figure 3a), sheer number of instances per image (Supplementary Figure 3b), and range of confluences represented (Supplementary Figure 2), LIVECell reaches the limit of what is distinguishable by eye in label-free 10X magnification phase contrast microscopy images.

Supplementary Table 1. Summary statistics of unique images in the LIVECell-dataset.

	**Total**		**Training**		**Validation**		**Test**	
	Images	Cells	Images	Cells	Images	Cells	Images	Cells
**A172**	608	131841	388	83175	68	15688	152	32978
**BT-474**	672	134542	429	85713	75	15088	168	33741
**BV-2**	608	383339	386	245638	70	45817	152	91884
**Huh7**	600	40882	352	24009	48	3886	200	12987
**MCF7**	735	353795	469	216052	82	39872	184	97871
**SH-SY5Y**	704	274726	449	168791	79	28796	176	77139
**SkBr3**	704	264816	449	169366	79	29693	176	65757
**SK-OV-3**	608	102411	266	41363	38	5531	304	55517
**Total**	**5239**	**1686352**	**3188**	**1034107**	**539**	**184371**	**1512**	**467874**

Supplementary Table 2. Summary of duplicates annotations in LIVECell

	**Total**	**Training**	**Validation**	**Test**	**Training Validation**
**Huh7**	68	38	1	16	17
**SK-OV-3**	72	27	0	36	13
**Total**	**144**	**65**	**1**	**52**	**30**

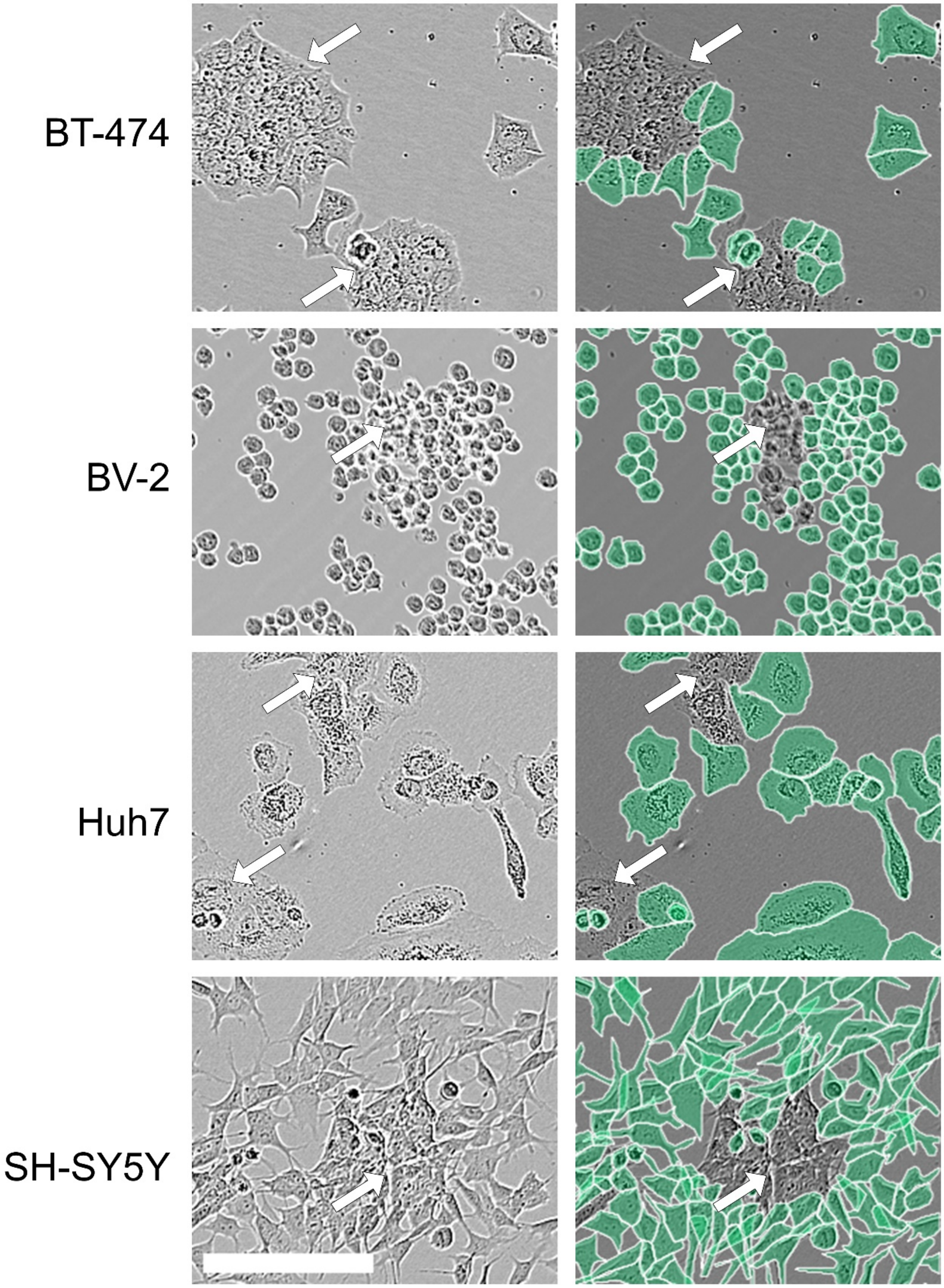

Supplementary Figure 1. Illustrative examples of cluster-forming cell types. Left panels show original phase contrast images, right panels show annotations overlaid in green. Arrows indicate clusters purposefully left unannotated due to ambiguous cell boundaries. Scale bar is 150μm and applies to all images. Each experiment is performed once.

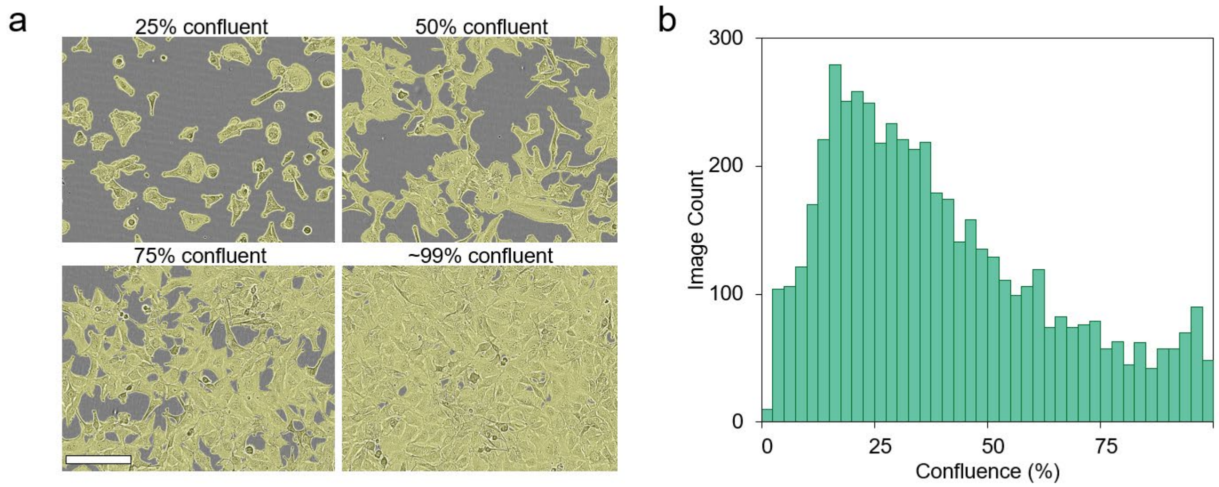

Supplementary Figure 2. Distribution of confluency in LIVECell. (a) Illustrative example showing different degrees of confluency. The grayscale phase contrast image is falsely colored by a yellow confluence mask that covers all pixels covered by cell mass according to manual annotation. Scale bar = 200 µm, which applies to all images in figure (b) Histogram showing distribution of per image confluence (%) calculated over all images in LIVECell. Each experiment is performed once. Scale bar is 200 µm and applies to all subfigures.

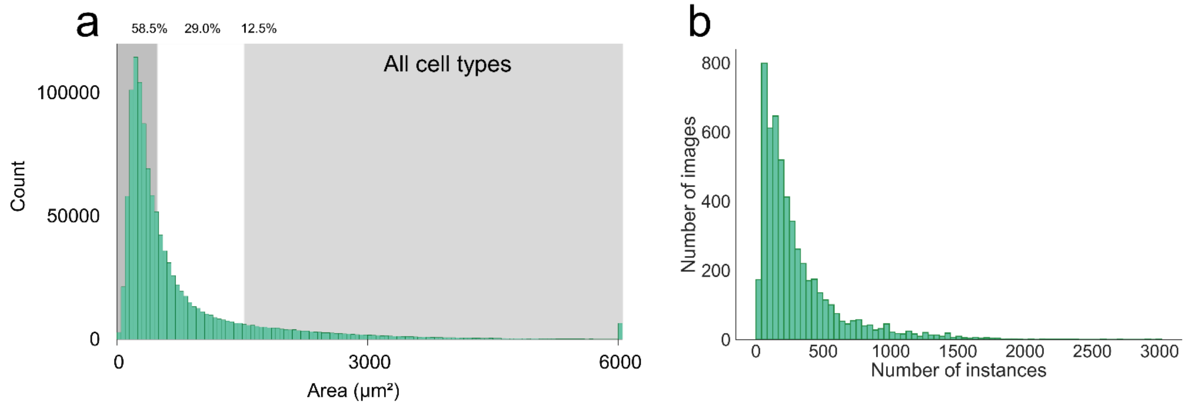

Supplementary Figure 3. Histograms showing distribution of cell size in µm² (a) and number of instances per image (b) calculated over all images in LIVECell. In a, the vertical color panes indicate the splits of cell size categories used for model evaluation and the percentages above each pane indicate how much of each cell type belong to each size category. The left-hand grey pane indicates small cells, the middle white pane medium sized cells and the right-hand grey pane indicate large cells.

Supplementary Note: Qualitative instance segmentation performance on LIVECell

To demonstrate the performance of our LIVECell-trained anchor-based and anchor-free models, we present image examples from our test dataset for each of the 8 cell types represented in LIVECell (Supplementary Figure 4).

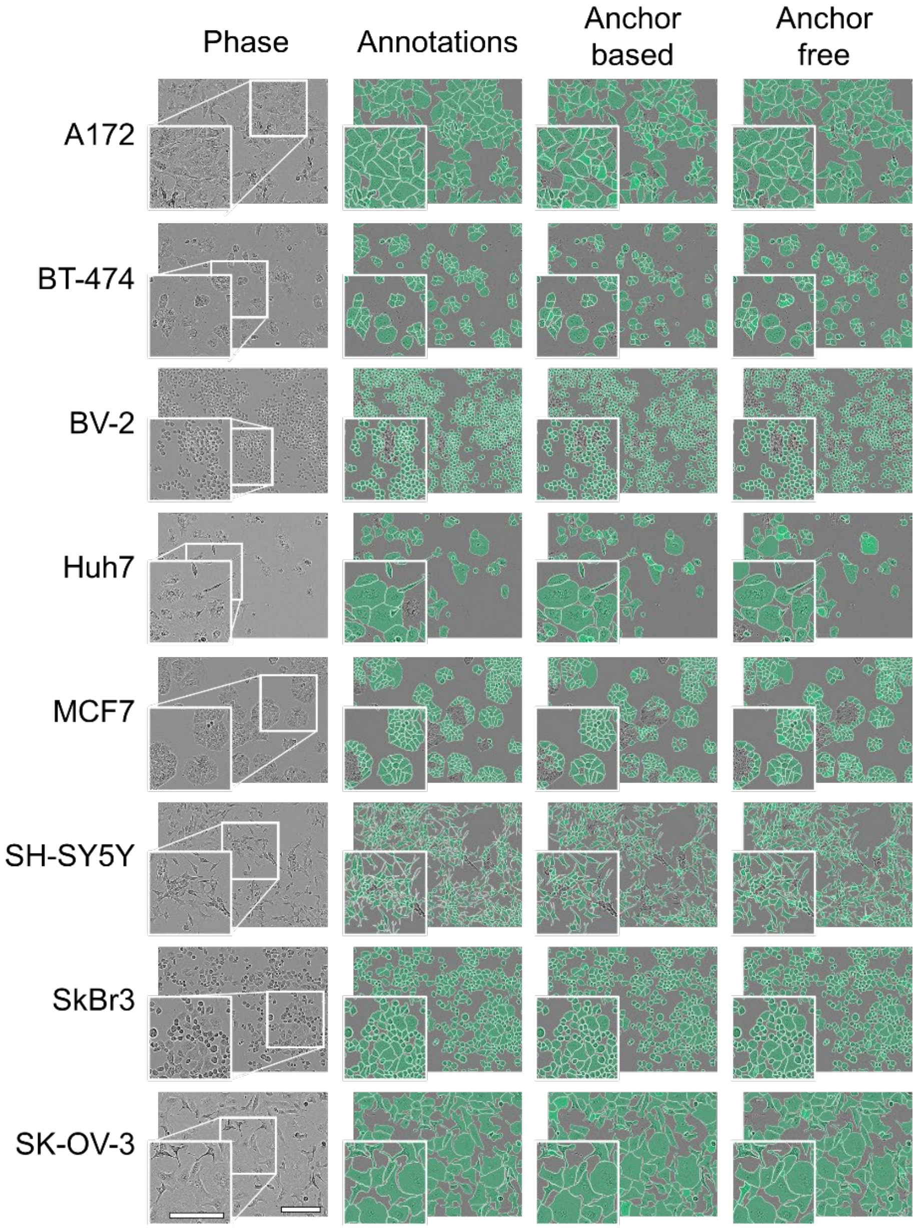

Supplementary Figure 4. Instance segmentation results from LIVECell trained models. For each of the eight LIVECell cell types, we show the phase contrast image (left), the manually-annotated cell instances (middle-left), the cell instances output from the anchor-based model (middle-right), and the cell instances output from the anchor-free model (right). Scale bar is 200 μm and applies to all images. Each experiment is performed once.

Supplementary Note: Evaluation metrics across different Intersection Over Union thresholds

In the main text, Figure 3 shows average precision (AP) and average false negative ratio (AFNR) for both the LIVECell-wide and single cell-type train and evaluate benchmarks. Here, we further dissect those metrics by plotting across different intersection over union (IoU) thresholds. In Supplementary Figure 5, each line in the plot corresponds to one evaluation dataset with the anchor-free (a) and anchor-based (b) models trained on the full LIVECell dataset. Furthermore, the false negative ratios (FNR) are plotted over each IoU threshold to give insight into how accurately the anchor-free (c) and anchor-based (d) model delineate the individual cell segmentation masks. Analogous plots were generated for models trained and evaluated on a single cell type (Supplementary Figure 6), with precision per IOU for anchor-free (a) and anchor-based (b) models, as well as FNR per IoU for anchor-free (c) and anchor-based (d). For the raw metrics, see Supplementary Table 8.

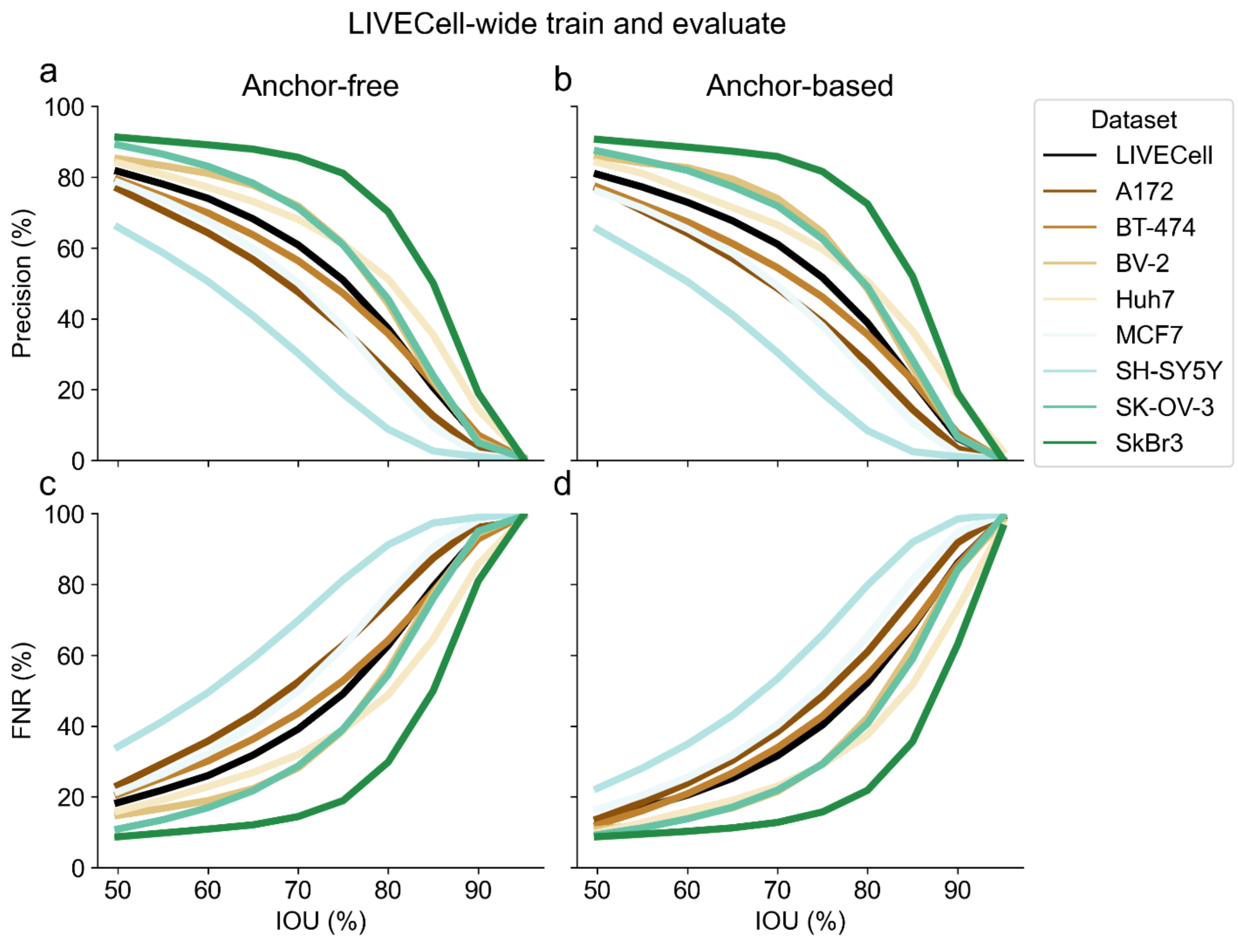

Supplementary Figure 5. Precision and False Negative Ratio (FNR) per Intersection over Union (IoU) level for a correct classification for the Anchor-free and Anchor-based models train on all LIVECell data. Both models are evaluated on LIVECell and each celltype subset. Precision for the anchor-free (a) and anchor-based (b) model are plotted against each IoU level, as well as FNR for the anchor-free(c) and anchor-based (d) models per subset.

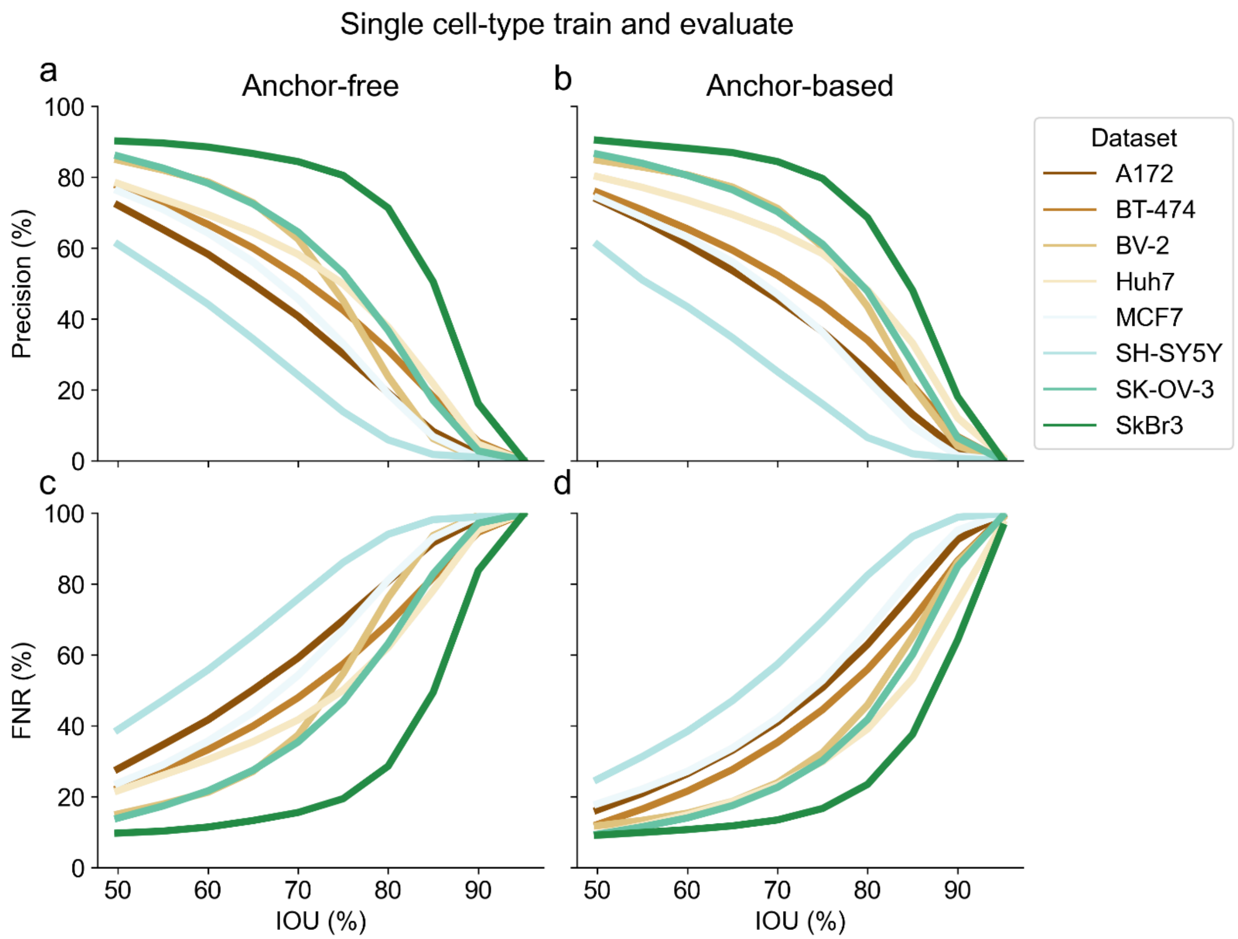

Supplementary Figure 6. Precision and False Negative Ratio (FNR) per Intersection of Union (IoU) level for a correct classification for Anchor-free and Anchor-based models train on all different cell types. All models are evaluated on the cell type it was trained on. Precision for the anchor-free (a) and anchor-based (b) models are plotted against each IoU level, as well as FNR for the anchor-free(c) and anchor-based (d) models per subset.

Supplementary Note: Detailed description of fluorescence-based nuclear counts

In Figure 4, we present data directly comparing the cell counts based on fluorescently labeled nuclei to label-free counts generated by our anchor-based and anchor-free models. Supplementary movies 1 and 2 show the segmentation results over the entire time course of the cell culture experiment for one example of each cell type. Supplementary Figure 7 shows select frames for the A172 (top) and A549 cells (bottom) alongside the fluorescent images and segmentation masks of nuclei.

Segmenting fluorescently labeled nuclei has been the gold standard method for counting cells for decades. While counting nuclei is generally an accurate measure, it is not without caveats. For live-cell imaging, the fluorescent labeling of nuclei requires either transient or stable transformation of cells, neither of which guarantee 100% of cells will express the fluorescent protein. Beyond just expression, cells along the edge of the image may not always be accurately counted via its nucleus, i.e., a cell's nuclei may exist out-of-frame while some other portion of its body is within field-of-view. These factors may artificially reduce nuclei counts relative to the true cell count. Other the other hand, certain cell types (e.g., cancer cells, muscle cells) may have multiple nuclei in a single cell, which can increase nuclei counts relative to the true cell count.

To investigate the extent to which the confounding factors inherent to fluorescent nuclear counts might be impacting our results, we quantified the percentage of unlabeled and multi-nuclear cells in an image of the A549 and A172 cells. Using the instance segmentation masks output from the anchor-free model, we performed overlap analysis with the nuclear segmentation masks. For A549 cells (Supplementary Figure 8, left panel), we found that 8.3% of A549 cells were unlabeled (cyan outlines), which includes cells along the edge whose nucleus may exist out-of-frame (arrows), and 0.4% of cells had multiple nuclei (magenta). A172 cells (Supplementary Figure 8, right panel) had a higher prevalence of multi-nucleated cells at 8.7% but less unlabeled cells at 4.2%. While these confounding factors do not impact the conclusions from our data in Figure 4,

they are important to note when considering any fluorescently labeled nuclei-based count and further underscore the importance of developing label-free solutions for counting cells.

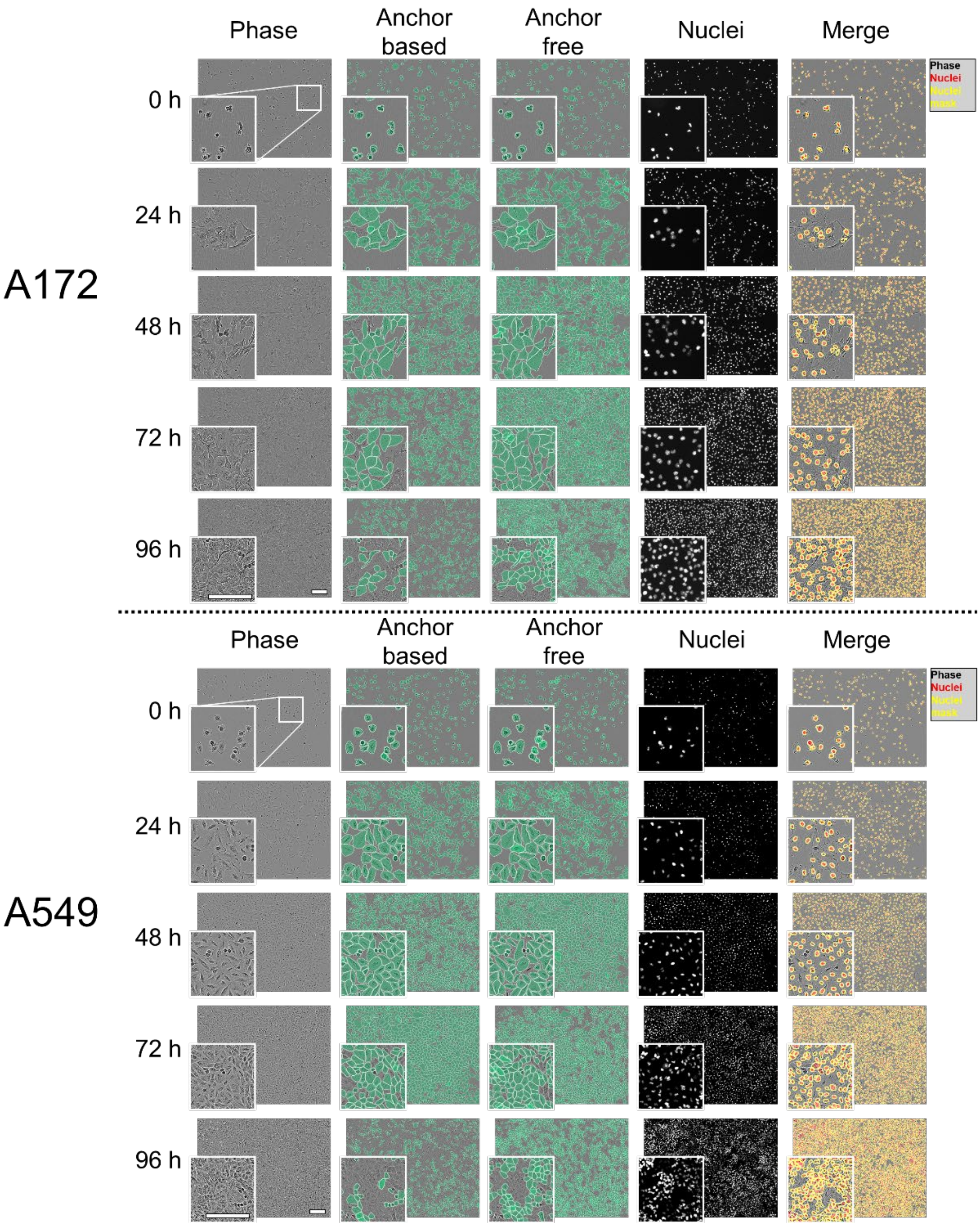

Supplementary Figure 7. Visualization of label-free and fluorescence-based segmentations for cell counting. Phase images and accompanying instance segmentations from the anchor-based and anchor-free models shown on the left, fluorescent nuclei and merged phase, nuclei, and nuclei segmentation masks shown on the right. Scale bar = 200 μm, which applies to all images within figure. Each experiment is performed once.

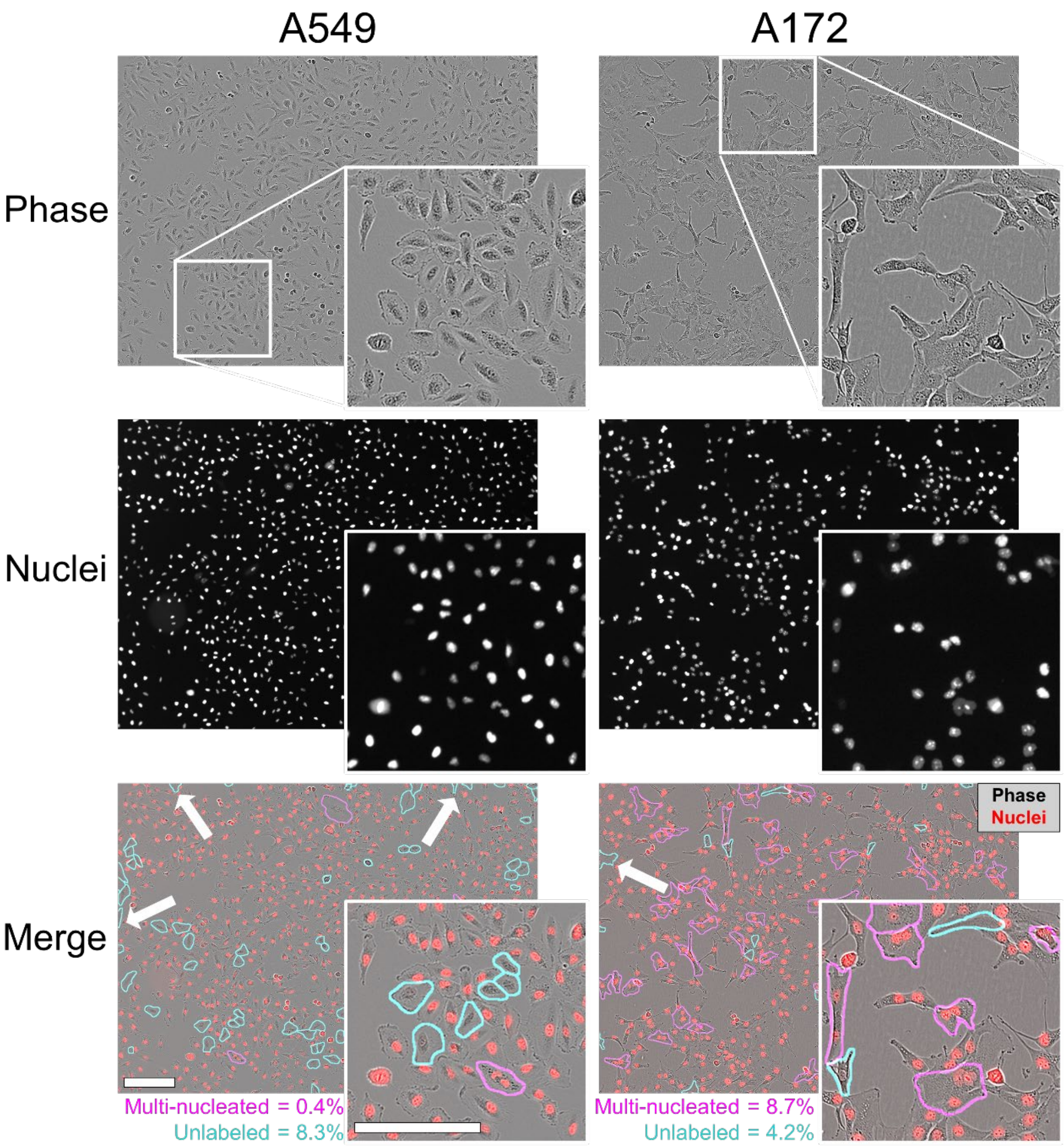

Supplementary Figure 8. Visualization of caveats to fluorescent nuclei counts. Phase contrast images (top), fluorescent nuclei (center) and merged images (bottom) for A549 (left) and A172 cells. Factors that may influence the accuracy of fluorescent nuclei-based cell counts are highlighted in the merged images, including unlabeled cells (cyan), cells without nuclei in frame (cyan, white arrows) and cells with multiple nuclei (magenta). Scale bar = 200 µm, which applies to all images within figure. Each experiment is performed once.

Supplementary Note: Model training

This supplementary note contains detailed information on training configuration and model training convergence. Supplementary Table 3 lists training parameters used to train the CNN-models used in this work. Supplementary Figure 9 and 10 show training loss curves during training on LIVECell for all benchmarks for the anchor-free and anchor-based model respectively.

Supplementary Table 3. Training parameters used to train instance segmentation CNN-models.

			**Training parameters**			
**Model**	**Setup**	**Cell type**	**Learning rate**	**Max iter.**	**Checkpoint**	**Warmup iter.**
**Anchor-based**	**All Cells**	ALL	0.02	5k	4.5k	1k
	**Single Cell**	A172	0.02	1.5k	1k	1k
		BT-474	0.02	1.5k	0.8k	1k
		BV-2	0.02	1.5k	1.2k	1k
		Huh7	0.02	1.5k	0.5k	1k
		MCF7	0.02	1.5k	1.4k	1k
		SH-SY5Y	0.02	1.5k	1.4k	1k
		SkBr3	0.02	1.5k	1.2k	1k
		SK-OV-3	0.02	1.5k	0.7k	1k
**Anchor-free**	**All Cells**	ALL	0.01	100k	32.5k	1k
	**Single Cell**	A172	0.01	10k	4.2k	1k
		BT-474	0.01	10k	6.2k	1k
		BV-2	0.01	10k	5.8k	1k
		Huh7	0.01	10k	5.0k	1k
		MCF7	0.01	10k	6.8k	1k
		SH-SY5Y	0.01	20k	11.4k	5k
		SkBr3	0.01	10k	14.6k	1k
		SK-OV-3	0.01	10k	7.0k	1k

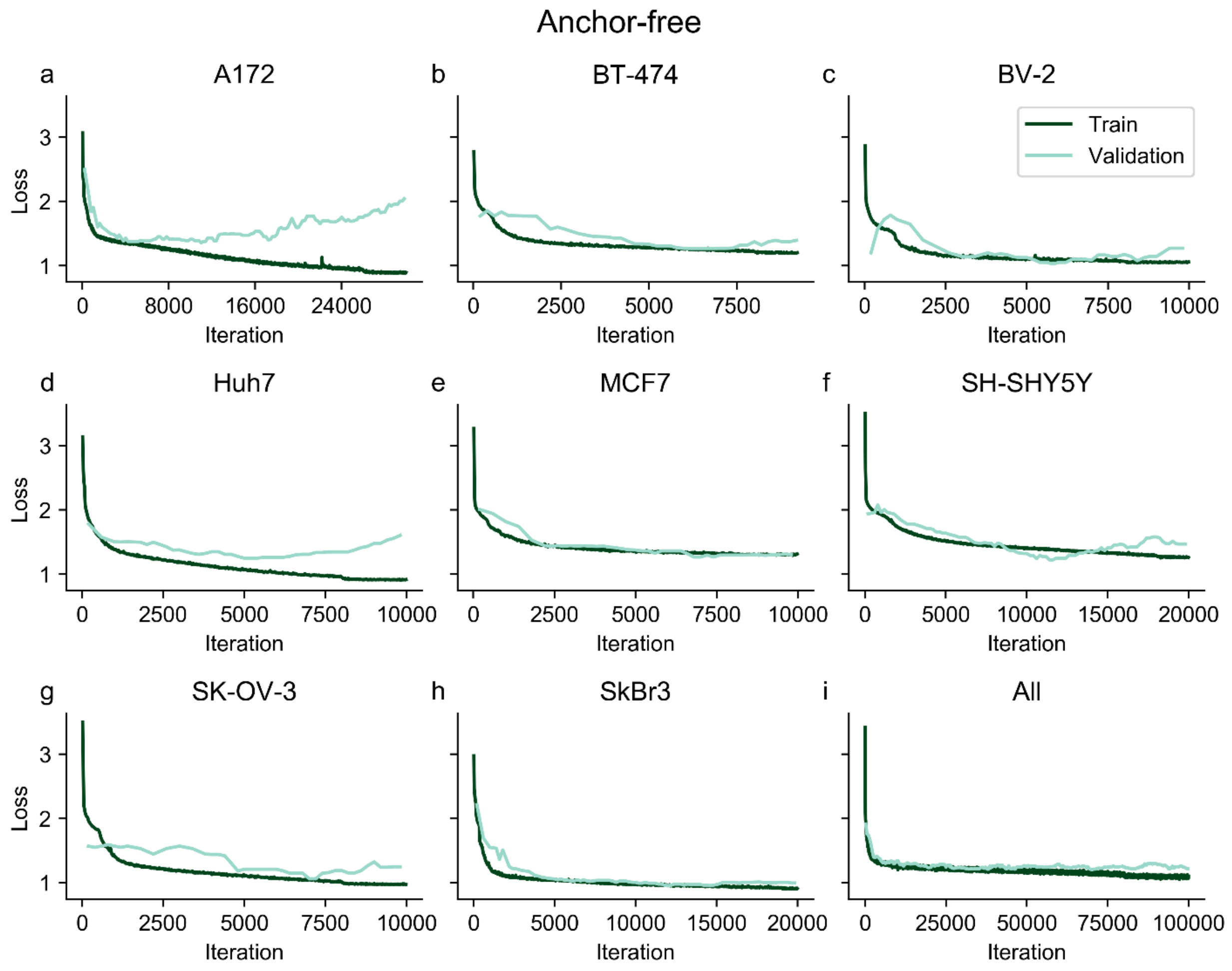

Supplementary Figure 9. Line plots of training and validation losses for anchor-free model training for single cell training (a-h) and training on whole dataset (i).

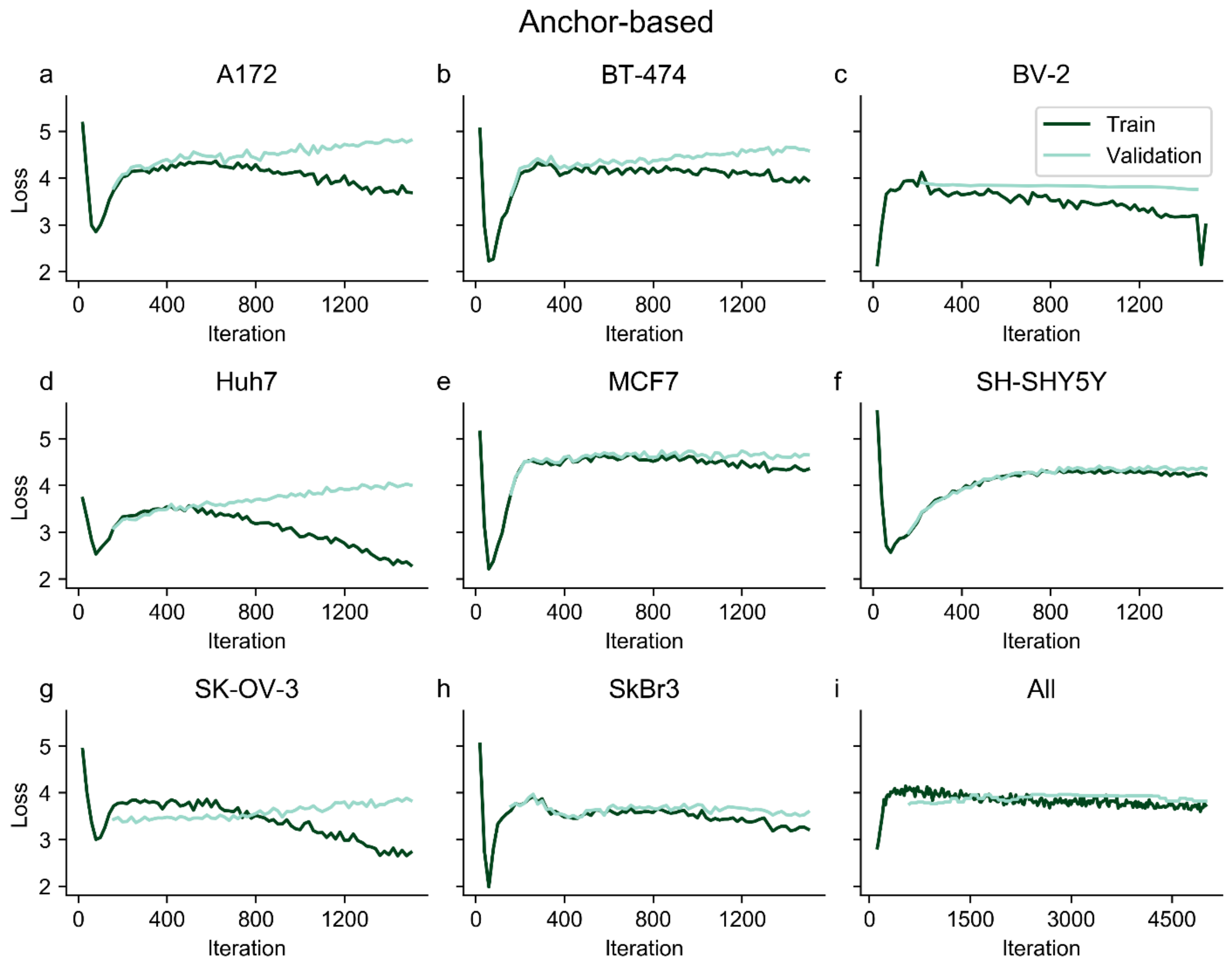

Supplementary Figure 10. Line plots of training and validation losses for anchor-based model training for single cell training (a-h) and training on whole dataset (i).

Supplementary Note: Computational performance

To understand the computational requirements to use our trained models, we traced the processing time and maximum memory usage during our validation experiment (Supplementary Figure 11, see segmentation performance in Figure 3). All inference was run on a workstation with an NVIDIA GeForce RTX 2080 Ti with 11 GB GPU RAM, Intel Xeon W-2145 CPU (8 cores, 3.7 GHz) and 64 GB RAM. The anchor-free model (Supplementary Figure 11a) processing time showed a piece-wise linear relationship with the number of objects, with discontinuity when memory use reaches its maximum around 1100 cells per image, and was able to predict close to 3000 cells in less than two seconds. The anchor-based model (Supplementary Figure 11b) showed a similar piece-wise linear relationship, although difficult to assess due to very few predictions above 1100 cells per image. Based on the observed relationships, the anchor-free model has lower intercept but steeper time evolution of objects over time compared to the anchor-based model. Due to the anchor-based model's few predictions over 1100 cells per image, we will not extrapolate to speculate regarding a possible point of break-even between the two models.

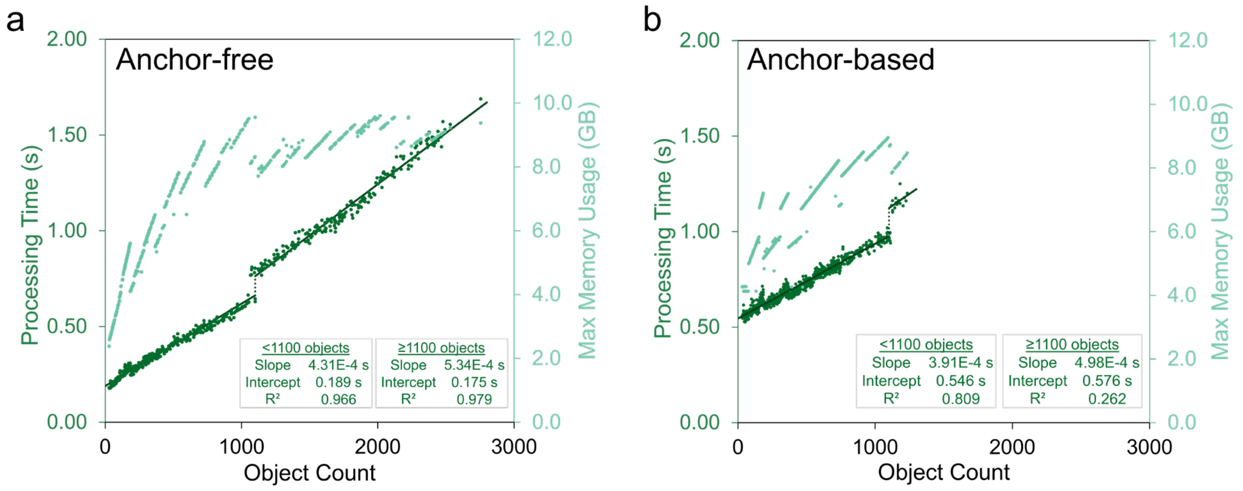

Supplementary Figure 11. Processing time (s) and max memory usage (GB) per image during inference for the anchor-free (a) and anchor-based (b) model.

Supplementary Note: Digital preprocessing for zero-shot inference to other microscopy images

As described in main text, LIVECell consists of Incucyte® HD phase contrast images acquired on an Incucyte® S3. To explore the ability to apply LIVECell trained models to perform inference on other phase microscopy images, we evaluated our LIVECell-trained anchor-based and anchor-free models on the EVICAN[10] and Cellpose[11] evaluation datasets. The EVICAN evaluation dataset contains 98 brightfield or conventional Zernike phase contrast images, which often have different imaging artefacts associated with them. For example, other light microscopy images are often characterized by uneven illumination patterns across the image. Zernike phase contrast and differential interference contrast (DIC) images typically have strong halo artefacts, causing extreme intensity shifts around cells. Additionally, the EVICAN evaluation dataset contains images acquired at 10X, 20X and 40X magnifications while LIVECell consists of only 10X images. The Cellpose evaluation dataset contains a mixture of 55 brightfield and fluorescence microscopic images, as well as 12 non-cellular images of repeated objects.

Because of these reasons, direct application of a LIVECell-trained model would yield unsatisfactory results; therefore, we developed a preprocessing pipeline and applied it to the entire EVICAN and Cellpose datasets prior to model inference. This pipeline includes:

1. In the case of fluorescence images, pixel intensity is first inverted.
2. Normalizing the median brightness of the image to 128,
3. Downsampling the image to match a 10X magnification (e.g., downsampling a 20X image by 50%),
4. Performing a large kernel median filter to estimate the background illumination pattern and dividing out that pattern,

5. Clipping intensity values to the range [70, 180], in order to remove the effects of Zernike halos,

Using this preprocessing scheme, we found LIVECell-trained models were able to achieve very impressive results on the EVICAN test dataset (Supplementary Figure 12a, Supplementary Table 4). The anchor-free model achieved an mAP score of 36.7, 24.2 and 11.4% for the easy, medium and difficult evaluations datasets of EVICAN, respectively (Supplementary Figure 12b). Surprisingly, the anchor-based model produced very impressive mAP scores, at 59.6, 32.5 and 22.2% for the easy, medium and hard evaluation datasets (Supplementary Figure 12b). We note that the EVICAN authors only report performance for segmenting cells and nuclei simultaneously, whereas we perform cell segmentation exclusively. To allow for a fair comparison, we replicated the original EVICAN Mask R-CNN (EVICAN-MRCNN) model trained on the EVICAN training set and evaluated it on all partitions of the test set and to reproduce previously reported results[10] (joint cell- and nucleus-segmentation AP50=61.6%). When considering only cell segmentation, both of our models surpass the EVICAN-MRCNN by a substantial margin, with the anchor-based model achieving over a two-fold higher mAP score than EVICAN-MRCNN (Supplementary Table 4). These results confirm that models trained on LIVECell transfer to other datasets out-of-the-box given appropriate preprocessing. However, it should be noted that the standard deviations for AP scores across images are quite high (Supplementary Figure 12b), which is similar to the large standard deviations reported by the EVICAN authors. This implies that although this preprocessing pipeline can enable LIVECell-trained models to successfully segment cells in many other types of light microscopy images, it is not without limitations. Future investigation will undoubtedly shed light on these limitations, as well as investigate the use of this type of preprocessing in LIVECell transfer learning applications.

Similarly, we found that LIVECell-trained models transferred to Cellpose generalist benchmark as well, showing non-trivial results, even on fluorescence images (Supplementary Figure 13a). This

is a pleasant surprise considering that LIVECell consists purely of phase contrast microscopy images but indicate that the scale and diversity of LIVECell enable models to learn general visual features of cells that transfer to other microscopy modalities as well. Overall, the LIVECell-trained models are not to be considered state-of-the-art on Cellpose (Segmentation AP50=77%, Cellpose generalist model[11]) but they perform on par with one of the baseline models trained on the Cellpose generalist data (Supplementary Figure 13b and c. Segmentation AP50 AP50=48.9% for the anchor-based model, and AP50=50.4 % for the anchor-free one, compared to AP50≈50% for Cellpose U-net3[11], see reference figure 4g). Similar to the EVICAN experiment, we note that the standard deviations of Cellpose segmentation are quite high (Supplementary Figure 13b) for the same reason. Even though the LIVECell-trained models segment many images well, there are cases where segmentation fails completely. This indicates the need for further research into how LIVECell-pretrained models can benefit other imaging modalities through the use of transfer learning.

Our preprocessing pipeline was implemented using opencv-python v4.5.1.48 and numpy 1.19.2 and average precision scores were calculated using pycocotools v2.0. The preprocessing script is available at https://github.com/sartorius-research/LIVECell.

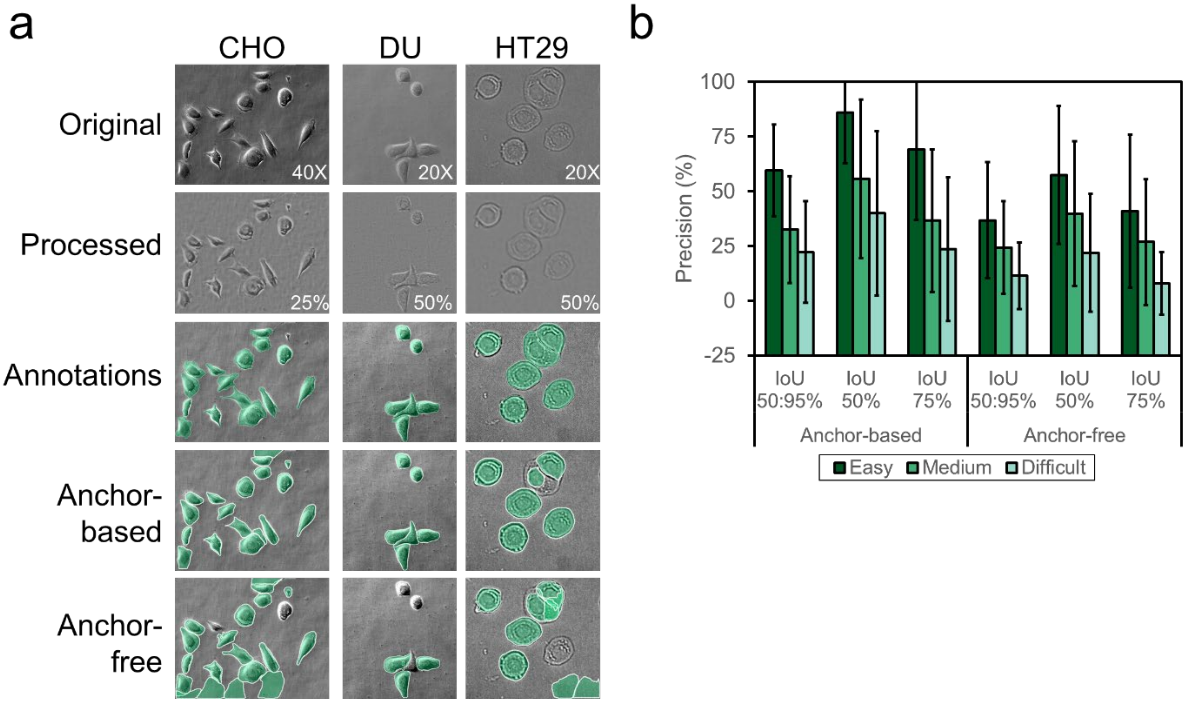

Supplementary Figure 12. Results of applying LIVECell-trained models to EVICAN images after proposed preprocessing. (a) Example images from the EVICAN evaluation dataset, including the original images, after processing, the EVICAN annotations, and LIVECell-trained model segmentation results. (b) Precision scores averaged across the entire EVICAN easy, medium and difficult evaluation datasets. Error bars indicate ± SEM, where n=33, 33 and 32 images for the Easy, Medium and Difficult EVICAN evaluation datasets, respectively. Each experiment is performed once.

Supplementary Table 4. Cell-segmentation results on EVICAN test set of EVICAN-MRCNN trained on the EVICAN training set and our anchor-free and -based model trained on LIVECell and applied to EVICAN in a zero-shot fashion after digital preprocessing. All scores are reported in %.

	**EVICAN-MRCNN**			**LIVECell Anchor-free**			**LIVECell Anchor-based**		
	mAP	AP50	AP75	mAP	AP50	AP75	mAP	AP50	AP75
Easy	24.6	50.0	22.0	36.7	57.4	40.9	59.6	86.1	69.0
Med	3.2	7.6	2.4	24.2	39.8	26.8	32.5	55.7	36.6
Difficult	4.7	12.9	1.7	11.4	22.0	7.9	22.2	39.9	23.6

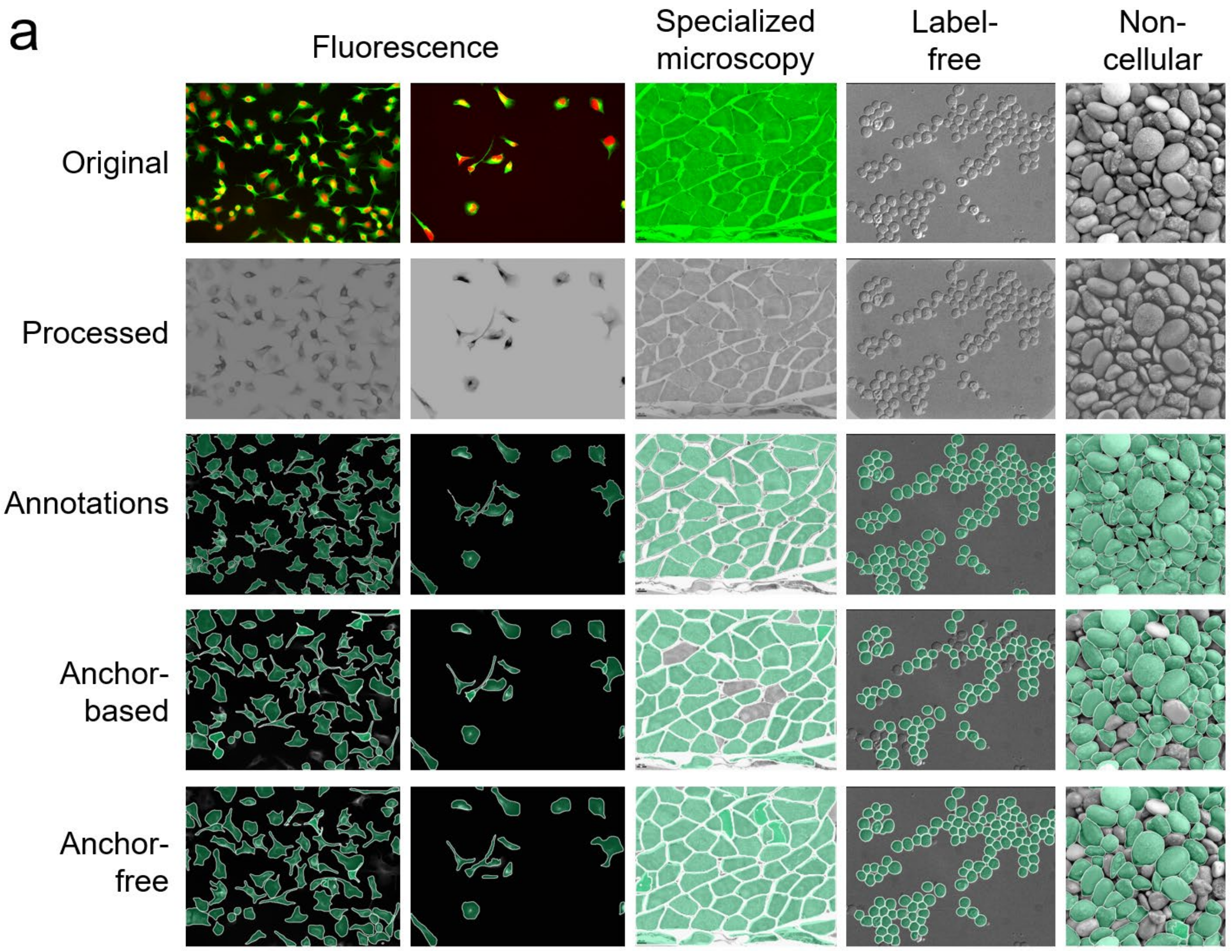

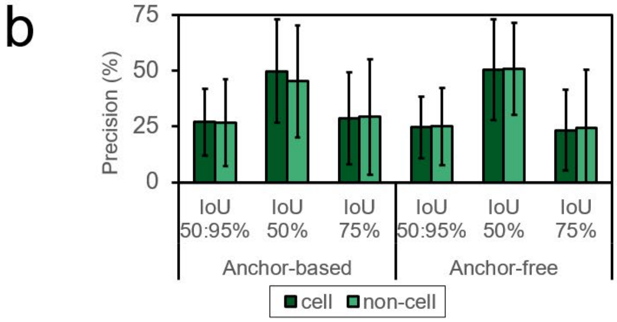

c

	Anchor-based			Anchor-free		
	50:95%	50%	75%	50:95%	50%	75%
All	26.9	48.9	28.6	24.5	50.4	23.3
Cell	26.9	49.7	28.6	24.5	50.3	23.3
Non-cell	26.7	45.2	29.2	24.9	50.9	24.4

Supplementary Figure 13. Results of applying LIVECell-trained models to Cellpose images after proposed preprocessing. (a) Example images from the Cellpose generalist evaluation dataset, including the original images, after processing, the Cellpose annotations, and LIVECell-trained model segmentation results. (b) Precision scores averaged across Cellpose cell and non-cell evaluation datasets. Error bars indicate ± SEM, where n = 55 and 13 images composing the cell and non-cell datasets.(c) Precision scores, mAP, AP50 and AP75, of LIVECell-trained model segmentation results on the Cellpose generalist benchmark as well as split according to cell and non-cell images. Each experiment is performed once.

Supplementary Note: Zero-shot inference of Cellpose generalist model to LIVECell

To assess to what extent the performance of models trained on LIVECell and Cellpose[11] overlap, we evaluated the Cellpose generalist models on all images in the LIVECell test-set (Supplementary Figure 14). We note that the Cellpose model generalize to LIVECell to less extent than what LIVECell-trained model generalize to Cellpose (Cellpose AP50=33% on LIVECell, see Supplementary Figure 14c, compared to the LIVECell-trained Anchor-free model AP50=50.4% on Cellpose, see Supplementary Figure 13c), even after fine-tuning the Cellpose cell diameter parameter. This might be due LIVECell's inclusion of far more cells per image than Cellpose, or that the chosen cell lines for LIVECell were specifically chosen to represent diverse morphology (Figure 1), both of which may allow models to learn more general features of cell appearance. While the Cellpose model generated reasonable segmentation masks for most low confluency images from early experiment timepoints, it struggled with high confluence images as well as certain cell types, particularly the two cell lines that tend to grow in rafts MCF-7 and BT-474 (Supplementary Figure 14a). Interestingly enough, the Cellpose-trained model achieved impressive performance on SkBr3 cells (AP=40.1% and AP50=82.1%, Supplementary Figure 14c), which confirm our results that SkBr3-cells are easier to segment compared to the other cell lines included in LIVECell (Figure 3). Similar to the experiments transferring LIVECell-trained models to EVICAN[10] and Cellpose (Supplementary Note: Digital preprocessing for zero-shot inference to other microscopy images), we observe large standard deviations performing zero-shot transfer of the Cellpose-models to LIVECell (Supplementary Figure 14b) due to a somewhat hit-and-miss model performance where some images are segmented quite well while others fail completely. To conclude, these results demonstrate that LIVECell fills a gap in the field that Cellpose cannot. Rather, we suggest that these diverse datasets provide complementary

resources for microscopic image analysis.

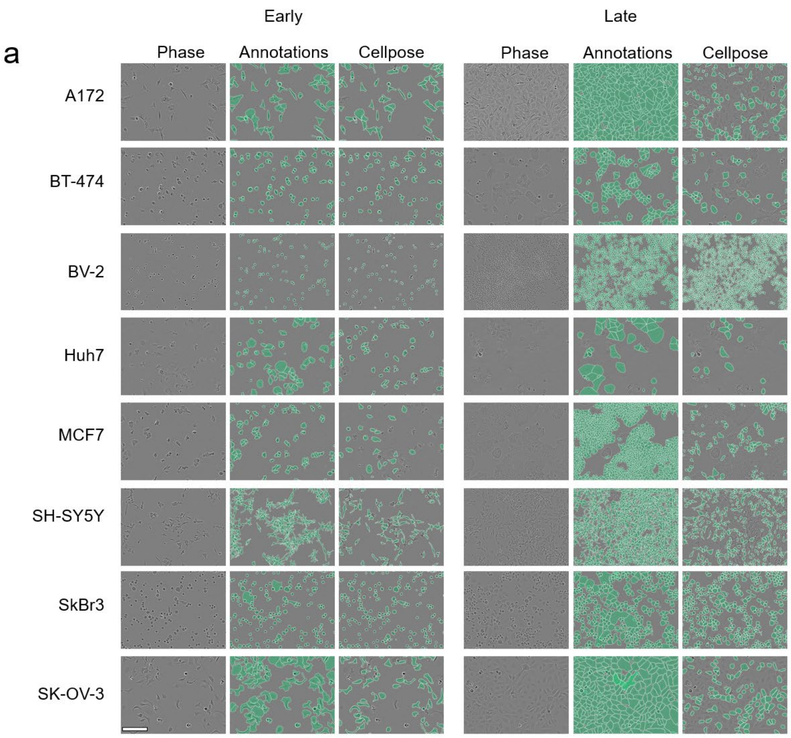

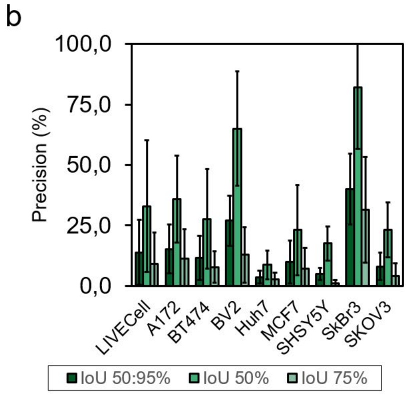

c

	IoU 50:95%	IoU 50%	IoU 75%
LIVECell	13.9	33.0	9.0
A172	15.3	35.9	11.4
BT-474	11.6	27.6	7.8
BV-2	27.0	65.0	12.8
Huh7	3.7	8.7	2.7
MCF7	9.9	23.2	7.2
SH-SY5Y	4.9	17.6	1.2
SkBr3	40.1	82.1	31.5
SK-OV-3	8.1	23.3	4.1

Supplementary Figure 14. Results of applying the Cellpose generalist model to the LIVECel test datasetl. (a) Example images from each cell type in LIVECell from early and late time points, including the original phase contrast images, the LIVECell annotations, and the Cellpose trained model segmentation results. Scale bar = 200 µm, which applies to all images in figure. (b) Precision scores averaged across each cell type in the LIVECell test set. Error bars indicate ± SEM, where n for each cell type is equal to the number of images in each test dataset as outlined in Supplementary Table 1. (c) Precision scores, mAP, AP50 and AP75, of the Cellpose generalist model segmentation results on the LIVECell test set as well as split according to cell type. Scale bar is 200 µm and applies to all subfigures.

References

1. Cai, Z. & Vasconcelos, N. Cascade R-CNN: High Quality Object Detection and Instance Segmentation. *IEEE Transactions on Pattern Analysis and Machine Intelligence* 1–1 (2019) doi:10.1109/TPAMI.2019.2956516.
2. Zhang, H. *et al.* ResNeSt: Split-Attention Networks. *arXiv:2004.08955 [cs]* (2020).
3. Zhang, H. *et al.* chongruo/detectron2-ResNeSt. https://github.com/chongruo/detectron2-ResNeSt (2020).
4. Lee, Y. & Park, J. CenterMask: Real-Time Anchor-Free Instance Segmentation. *Proceedings of the IEEE/CVF Conference on Computer Vision and Pattern Recognition (CVPR)* 13906–13915 (2020).
5. Lee, Y. & Park, J. youngwanLEE/centermask2. https://github.com/youngwanLEE/centermask2 (2020).
6. Tian, Z., Shen, C., Chen, H. & He, T. FCOS: Fully Convolutional One-Stage Object Detection. *Proceedings of the IEEE/CVF International Conference on Computer Vision (ICCV)* 9627–9636 (2019).
7. Lin, T.-Y. *et al.* Microsoft COCO: Common Objects in Context. in *Computer Vision – ECCV 2014* (eds. Fleet, D., Pajdla, T., Schiele, B. & Tuytelaars, T.) 740–755 (Springer International Publishing, 2014). doi:10.1007/978-3-319-10602-1_48.
8. Paszke, A. *et al.* PyTorch: An Imperative Style, High-Performance Deep Learning Library. *Advances in Neural Information Processing Systems 32* 8024–8035 (2019).
9. Wu, Y., Kirillov, A., Massa, F., Lo, W.-Y. & Girshick, R. Detectron2. https://github.com/facebookresearch/detectron2 (2019).
10. Schwendy, M., Unger, R. E. & Parekh, S. H. EVICAN—a balanced dataset for algorithm development in cell and nucleus segmentation. *Bioinformatics* 36, 3863–3870 (2020).

11. Stringer, C., Wang, T., Michaelos, M. & Pachitariu, M. Cellpose: a generalist algorithm for cellular segmentation. *Nat. Methods* **18**, 100–106 (2021).
