# Supplementary material for: LIVECell—A large-scale dataset for label-free live cell segmentation
Source: Nat Methods. 2021 Aug 30;18(9):1038–45. doi: 10.1038/s41592-021-01249-6 (PMC8440198; doi:10.1038/s41592-021-01249-6)
Supplement: Supplementary file 2 — Reporting Summary [file 41592_2021_1249_MOESM2_ESM.pdf]

nature research

Corresponding author(s): Rickard Sjögren

Last updated by author(s): Jul 2, 2021

Reporting Summary

Nature Research wishes to improve the reproducibility of the work that we publish. This form provides structure for consistency and transparency in reporting. For further information on Nature Research policies, see our Editorial Policies and the Editorial Policy Checklist.

Statistics

For all statistical analyses, confirm that the following items are present in the figure legend, table legend, main text, or Methods section.

n/a	Confirmed	
☐	☒	The exact sample size (*n*) for each experimental group/condition, given as a discrete number and unit of measurement
☐	☒	A statement on whether measurements were taken from distinct samples or whether the same sample was measured repeatedly
☐	☒	The statistical test(s) used AND whether they are one- or two-sided<br>*Only common tests should be described solely by name; describe more complex techniques in the Methods section.*
☐	☒	A description of all covariates tested
☐	☒	A description of any assumptions or corrections, such as tests of normality and adjustment for multiple comparisons
☐	☒	A full description of the statistical parameters including central tendency (e.g. means) or other basic estimates (e.g. regression coefficient) AND variation (e.g. standard deviation) or associated estimates of uncertainty (e.g. confidence intervals)
☐	☒	For null hypothesis testing, the test statistic (e.g. F, t, r) with confidence intervals, effect sizes, degrees of freedom and P value noted<br>*Give P values as exact values whenever suitable.*
☒	☐	For Bayesian analysis, information on the choice of priors and Markov chain Monte Carlo settings
☒	☐	For hierarchical and complex designs, identification of the appropriate level for tests and full reporting of outcomes
☒	☐	Estimates of effect sizes (e.g. Cohen's *d*, Pearson's *r*), indicating how they were calculated

Our web collection on statistics for biologists contains articles on many of the points above.

Software and code

Policy information about availability of computer code

Data collection	Images were divided into batches for upload using MODDE 12.1 (Sartorius Data Analytics)<br>Images were annotated by outlining them with polygons using a commercially available, cloud-based, annotation software (V7Labs Darwin)
Data analysis	Code used for data analysis is publicly available at https://github.com/sartorius-research/LIVECell<br>Cell morphologies were quantified and analyzed using Python 3.6, opencv-python v4.4.0.46, scikit-image v0.17.2, SciPy v1.5.2, scikit-learn v0.22.2 and matplotlib v3.1.1.<br>CNN models for segmentation were trained using Python 3.6.10 with PyTorch v1.5.0, Detectron2 v0.2.1 and Python v3.7.7, PyTorch v1.5.0 and Detectron2 v0.3.<br>Segmentation benchmarks were evaluated using Pycocotools (https://github.com/cocodataset/cocoapi/tree/master/PythonAPI/pycocotools).<br>The cell type generalization benchmark was implemented in Python v3.6.7 and Pandas v1.1.5<br>The comparison to fluorescence based cell counts was implemented using Python v3.6.7, scikit-learn v0.24.1 and Scipy v1.3.1.<br>The image preprocessing scripts used for transfer to other datasets were implemented using Python 3.6, opencv-python v4.5.1.48, numpy 1.19.2 , and pycocotools v2.0.

For manuscripts utilizing custom algorithms or software that are central to the research but not yet described in published literature, software must be made available to editors and reviewers. We strongly encourage code deposition in a community repository (e.g. GitHub). See the Nature Research guidelines for submitting code & software for further information.

Data

Policy information about availability of data

All manuscripts must include a data availability statement. This statement should provide the following information, where applicable:

- Accession codes, unique identifiers, or web links for publicly available datasets
- A list of figures that have associated raw data
- A description of any restrictions on data availability

The LIVECell dataset, trained models and config files to apply the models have been deposited at https://sartorius-research.github.io/LIVECell/

Field-specific reporting

Please select the one below that is the best fit for your research. If you are not sure, read the appropriate sections before making your selection.

☒ Life sciences ☐ Behavioural & social sciences ☐ Ecological, evolutionary & environmental sciences

For a reference copy of the document with all sections, see nature.com/documents/nr-reporting-summary-flat.pdf

Life sciences study design

All studies must disclose on these points even when the disclosure is negative.

Sample size	At the beginning of the project, it was not exactly known how many images would be required to successfully train machine learning models. We chose a set of images representative of the problem we wanted to address and estimated that the resulting number of annotated cells would surpass 1 million based on average cell densities, which would put the resulting dataset on a similar scale as popular machine learning datasets such as Microsoft COCO.
Data exclusions	No data was excluded.
Replication	All images are being made available. The training, validation and test set splits used in this study are also being made available along with configuration files to train our models.
Randomization	The test set used to validate machine learning models was selected as one complete well at random per cell type before annotation started. The validation set used to monitor machine learning model training was selected as random images from the training set.
Blinding	Not relevant since no human subjects were involved in the study.

Reporting for specific materials, systems and methods

We require information from authors about some types of materials, experimental systems and methods used in many studies. Here, indicate whether each material, system or method listed is relevant to your study. If you are not sure if a list item applies to your research, read the appropriate section before selecting a response.

Materials & experimental systems

n/a	Involved in the study	
☒	☐	Antibodies
☐	☒	Eukaryotic cell lines
☒	☐	Palaeontology and archaeology
☒	☐	Animals and other organisms
☒	☐	Human research participants
☒	☐	Clinical data
☒	☐	Dual use research of concern

Methods

n/a	Involved in the study	
☒	☐	ChIP-seq
☒	☐	Flow cytometry
☒	☐	MRI-based neuroimaging

Eukaryotic cell lines

Policy information about cell lines

Cell line source(s)	A172, BT-474, MCF7, SH-SY5Y, SkBr3, SK-OV-3 were purchased from ATCC. Huh7 was purchased from CLS cell line services. BV-2 was purchased from Interlab Cell line collection.
Authentication	The cell lines were purchased from commercial sources and cam with paperwork to support the authenticity. The cell lines chosen are also standard with well documented expression profiles and have been studied extensively in other experiments. We did not do any further testing for authenticity.

Mycoplasma contamination	The commercial vendor supply the cell lines with mycoplasma status, which were all negative. We did not do any further testing but keep the cell lines in cultures for less than 3 months at a time to avoid any issues.
Commonly misidentified lines (See ICLAC register)	No commonly misidentified cell lines were used.
